# The “*Nasci Bem*” app for health professionals and families of newborns: construction, validation and evaluation *

**DOI:** 10.1590/1518-8345.7264.4260

**Published:** 2024-11-22

**Authors:** Ingrid Lucchese, Fernanda Garcia Bezerra Góes, Maithê de Carvalho e Lemos Goulart, Maria da Anunciação Silva, Aline Cerqueira Santos Santana da Silva, Liliane Faria da Silva

**Affiliations:** ^1^ Universidade Federal Fluminense, Instituto de Humanidades e Saúde, Rio das Ostras, RJ, Brazil.; ^2^ Scholarship holder at the Conselho Nacional de Desenvolvimento Científico e Tecnológico (CNPq), Brazil.; ^3^ Universidade Federal do Estado do Rio de Janeiro, Rio de Janeiro, RJ, Brazil.; ^4^ Universidade Federal Fluminense, Escola de Enfermagem Aurora de Afonso Costa, Niterói, RJ, Brazil.

**Keywords:** Mobile Applications, Rooming-in Care, Hospitals, Maternity, Infant, newborn, Delivery Rooms, Educational Technology

## Abstract

to construct, validate and evaluate an app for mobile devices on humanized care practices for newborns at habitual risk in maternity wards, aimed at health professionals and family members.

a methodological study in seven stages: Literature review; Content organization; App construction; Validation by experts; Suitability after validation; Evaluation by the target audience; and Suitability after evaluation. A total of 22 experts took part in the validation and 21 individuals from the target audience in the evaluation. The instruments addressed content, face and/or motivation, with the System Usability Scale for usability. The following was calculated: Concordance Index and Usability Score.

in the validation process, the app achieved a Concordance Index of 0.97; ranging from 0.82 to 1.0 across the items analyzed. In the evaluation, the index was 0.99, varying from 0.95 to 1.0. In usability of the first version, the Concordance Index was 0.92 and the Usability Score was 93. In the second version, the index was 0.93 and the score was 94, indicating better usability achieved since the first version.

the “*Nasci Bem*” app proved to be comprehensible, relevant, pertinent, efficient, easy to use, with low inconsistency, and with excellent potential to use it as educational technology for professionals and family members.

## Highlights

**(1)** The study constructed, validated and evaluated the “ *Nasci Bem*” mobile app.

**(2)** A total of 22 experts and 21 health professionals and family members took part in the survey.

**(3)** The validation and evaluation processes showed high agreement and usability levels.

**(4)** The educational technology is understandable, relevant, efficient and easy to use.

**(5)** The “*Nasci Bem*” app contributes to digital health.

## Introduction

 The Brazilian Prenatal and Birth Humanization Program aims at improving prenatal care and childbirth/puerperium care for mothers and newborns, promoting a humanized approach to childbirth as a truly human experience ^( 1 )^. According to the World Health Organization (WHO) global guidelines for the care of healthy pregnant women, immediate intervention by the health team in habitual risk situations is not recommended, emphasizing the uniqueness of each woman and her childbirth process for a positive experience. It is essential to involve them in decisions about the care they receive, even when professional interventions are necessary ^(2).^

 In Brazil, the predominant care model during childbirth is still technocratic, focused on health professionals and prone to inappropriate interventions ^(3)^. Therefore, humanized practices recommended by the WHO are not being effectively adopted, such as the following ones: early skin-to-skin contact, which encourages breastfeeding, prevents neonatal hypothermia and strengthens the bond between mother and baby; breastfeeding in the first hour of life, which reduces early weaning and promotes cardiorespiratory health; timely clamping of the umbilical cord, which reduces anemia in the first months of life; and the presence of a companion in the delivery room, recognized as promoting these good practices ^(1 - 3)^.

 There is a need to raise awareness among health professionals about humanized practices in maternity wards, with the aim of providing comfort and safety and reducing unnecessary C-sections ^(4)^, which make skin-to-skin contact and early breastfeeding difficult but not impossible, due to anesthesia and the zero-degree bed position ^(5)^. In addition, women undergoing a C-section release less oxytocin (a hormone that is crucial for childbirth) immediately after delivery ^(6)^ and experience more pain and discomfort after the surgery ^(7)^. However, the C-section rate in Brazil far exceeds those suggested by the WHO (from 10% to 15%) ^(8)^, standing at around 55% ^(9)^, which limits the effective application of humanized practices. However, the ideal practices for caring for a newborn after a C-section should be the same as for vaginal deliveries ^(4)^.

 Several factors favor adherence to humanized practices for newborns at habitual risk in maternity wards, especially vaginal delivery, good vitality and early skin-to-skin contact, which eases timely clamping of the umbilical cord and breastfeeding in the first hour of life ^(1 - 3)^. Although some neonates require interventions, practices such as separation of the dyad, use of inhaled oxygen, oronasopharyngeal, gastric and tracheal aspiration ^(1, 10)^ and early or immediate clamping of the umbilical cord ^(4)^ still persist in many hospital centers, even in healthy newborns ^(3)^. These interventions are sometimes preferred, even in the absence of justifiable pathological conditions ^(10)^. 

 Health professionals demonstrate knowledge of humanized practices, which is considered a fundamental starting point for their implementation, according to the literature ^(11)^. However, effective adherence to them requires sensitization of managers and professionals, institutional support and evidence-based updating. In addition, it is recommended to share guidance on safe postnatal assistance from prenatal care to the maternity-home transition ^(12)^, with the aim of training pregnant/puerperal women and family members. 

 Integrated into the patients’ and professionals’ routines, educational health technologies have the potential to promote health through information on the prevention and treatment of health problems ^(13)^. According to the 33 ^rd^ Annual Survey on the Use of Information Technology by the Getúlio Vargas Foundation, in 2022 Brazil had 447 million digital devices (computers, laptops, tablets and smartphones), more than two per inhabitant ^(14)^. Given the widespread presence of these devices, especially smartphones, using educational technologies is a promising resource for nurses to act dynamically as health educators. Globally, digital health is recognized as an effective tool for promoting health information, reaching diverse audiences ^(15 - 16)^. 

 In 2020 and in line with global recommendations, Brazil launched the 2020-2028 Digital Health Action Plan, outlining strategies to achieve the Digital Health Vision. This plan proposed creating an integrated platform to promote data exchange in the Health Care Network, with the expectation of implementing it in all 27 federative units by the end of 2023 ^(17)^. This development supports the design of digital health tools aimed at health education, easing continuous care regardless of time or place. 

 In the literature, some research studies have identified apps in this area: one of them for pregnant women in prenatal care, aimed at promoting health during the pregnancy and puerperal periods ^(13)^; and another one aimed at health professionals to assess risks to neonatal health and disseminate care practices for newborns ^(18)^. However, knowledge gaps were evident in the scarcity of international and national studies on apps addressing humanized care practices for newborns at habitual risk in maternity wards and aimed at professionals and family members simultaneously. This gave rise to the intention of producing a mobile app in this area, as an educational technology to support assertive decisions by health professionals and provide practical and accessible knowledge to family members from prenatal care onwards. 

This research aims at contributing to the advancement of scientific knowledge and digital health by fully presenting the process from conception to evaluation of an innovative app that brings together up-to-date content on newborn care practices (recommended, recommended with criteria, and not recommended), in a mobile, accessible and free-to-access way, which reinforces the relevance of this study.

Therefore, the study objective was to construct, validate and evaluate a mobile app on humanized care practices for newborns at habitual risk in maternity wards, aimed at health professionals and family members.

## Method

### Study design

 A methodological study with a quantitative approach, which constructed, validated and evaluated an educational technology in the mobile app format in seven stages: 1) Literature review; 2) Content organization; 3) App construction (design and development); 4) App validation by experts; 5) Suitability after validation; 6) App evaluation by the target audience; and 7) Suitability after evaluation. Methodological studies are generally non-experimental and focus on the creation of new educational materials ^(19 - 20)^. 

### Setting

The study took place in a virtual environment.

### Period

The app development process began in January 2023 and data collection for validation and evaluation took place between August and November 2023.

### Population

The study population included experts (health professionals with experience in Neonatology, Pediatrics or Obstetrics) and the target audience (health professionals from maternity hospitals and pregnant women, puerperal women, and families of newborns).

### Selection criteria

 The inclusion criteria used to select the experts wereas follows: health professionals with experience in Neonatology, Pediatrics or Obstetrics; and the Fehring criteria ^(20)^ were applied, which assign a score from 1 to 5 points, considering participation in scientific events in the last two years on the subject matter (1 point), at least five years’ experience in the area (2 points), publication in indexed journals on the subject matter (2 points), specialist degree (3 points), MSc (4 points) and Ph.D. (5 points). To check the score, the data in the Lattes Platform résumés of each selected professional were checked, excluding those that failed to reach the minimum score of five points. In addition, the exclusion criterion was professionals with exclusively administrative activities. 

The target audience was selected using the following inclusion criteria: health professionals working in the delivery room and/or in the rooming-in unit, pregnant women, puerperal women and family members (over the age of 18) of newborns, with Internet access. The exclusion criteria involved health professionals who worked as nursing assistants, who did not provide direct care to a mother-baby dyad or who were only involved in administrative activities. In addition, pregnant/women or family members of newborns with physical and/or mental limitations to answer online forms or who were illiterate were also excluded.

### Definition of the sample

 The participants were invited by convenience and non-probability selection. The sample consisted of 22 experts and 21 individuals from the target audience, who returned their evaluations within ten days. This distribution follows the recommendation in the literature on the development of educational technologies, which indicates a minimum of nine participants for each group of evaluators ^(21)^. Of the target audience, eight subjects were health professionals and 13 were pregnant women, puerperal women and family members of newborns. 

### Study variables

The first section of the instrument validated by the experts included closed questions to characterize the participants, such as age, gender, schooling and professional field, qualifications, and length of experience. The assessment instrument for the target audience included aspects such as age, gender, schooling level, profession, type of involvement (health professional or family member of a newborn), time working in the maternity ward (for health professionals) and relationship with the newborn (for family members). The second section dealt with specific questions related to the analysis of the educational technology.

### Instruments used to collect the information

 The data collection process for validating and evaluating the mobile app was conducted using *Google Forms* on the *Google* platform. The online survey page contained detailed information about the project, the Informed Consent Form (ICF) available for download by the participants and the electronic form for data collection. 

 The previously validated instrument for validation of the app by the experts included eight questions on content and seven on face. For the evaluation by the target audience, six questions on content, six on face and three on motivation were used, resorting to an instrument that had also been previously validated. The items were scored on a Likert scale from 1 to 4 points, as follows: “I strongly disagree” (1 point), “I somewhat disagree” (2 points), “I somewhat agree” (3 points) and “I strongly agree” (4 points) ^(22)^. 

 Sequentially, both groups of evaluators employed the System Usability Scale (SUS) ^(23)^, made up of ten items. This tool uses a score from 1 to 5 points, namely: “I strongly disagree” (1 point), “I somewhat disagree” (2 points), “I neither agree nor disagree” (3 points), “I somewhat agree” (4 points) and “I strongly agree” (5 points) ^(24)^. In addition, at the end of the instruments, a space was provided for suggestions and comments to improve the educational technology in health. 

### Data collection

 The first stage, a narrative literature review, aimed at identifying, gathering and synthesizing information on recommended, critically recommended and non-recommended care practices for the assistance to be provided to newborns at habitual risk in maternity wards. Based on authors that are experts in the field and on documents from the United Nations Children’s Fund (UNICEF), the WHO, the Brazilian Health Ministry (*Ministério da Saúde*, MS) and the Brazilian Society of Pediatrics (*Sociedade Brasileira de Pediatria*, SBP), the review searched for publications from the last ten years, including articles, manuals, guides, guidelines and resolutions related to the study subject matter. This search was carried out through direct consultation of the websites of these organizations, reinforcing reliability and timeliness of all the information collected, involving terms such as “Newborn”, “Maternity Hospital”, “Delivery Rooms” and “Rooming-In”. 

In the second stage, corresponding to content organization, the scientific evidence found in the literature was used to create an analysis matrix with the main findings on the websites of the scientific organizations cited. Based on the identification of these practices, the essential actions to be applied in the delivery room and the Rooming-In area were extracted, with the aim of promoting humanized care practices. Based on this organization, the team developed the content for the technology, including texts, images and links. The purpose was to create a roadmap to guide construction of the app in a creative, appealing, intuitive, detailed, motivating and effective way.

 In the third stage, referring to the mobile app creation, a first meeting was held between the study team and a developer specializing in Computer Science, who owns a service company in the area. It should be noted that the app’s development, a decisive step, must be carried out by specialized professionals under supervision of the researchers, as indicated by another study ^(25)^. During this initial meeting, ideas were discussed for the instructional design and navigation structure, adapted in the form of a free downloadable app. The interface was designed to ensure interactivity, autonomy and accessibility for all commands. 

A second meeting took place 23 days later, in which the developer presented a preliminary version of the app in Android Application Pack (APK) format. This version already contained the tabs with the educational material, as well as the predefined colors and fonts, allowing adjustments to be made according to the team’s preferences. Subsequently, a new data insertion stage was carried out in the educational tool, resulting in the creation of the interfaces: main menu, booklets, quiz and information on each user profile, which gave rise to the first version of the app.

In stages four and six, data were collected through invitations sent via WhatsApp, email and Instagram messages, inviting participants to validate or evaluate the mobile app. To preserve privacy, no identifiable contact lists were used. The invitation included a brief explanation of the project, identification of the researchers, a link to the research and the app in APK format for download on Android smartphones. A 10-day deadline was set for answers, with a mean of 30 minutes to analyze the app and fill in the form.

As the sample was non-probabilistic, consecutive and assembled over the period stipulated by the researchers, data collection went smoothly, with most of the experts and individuals from the target audience invited responding promptly to the material, without refusals or delays. It should be noted that, due to the nature of communication via social media, some of the invitations sent out were not answered, which is why more invitations were distributed than the minimum limit set: approximately 25 for the experts and 30 for the target audience.

It should be noted that the first version of the app was validated by the experts in stage four. Only after the suggested adaptations had been made in stage five did the second version proceed to be evaluated with the target audience in stage six. Thus, even after these phases, small changes were still necessary to prepare the final version of the app, which was completed in the seventh and final stage.

### Data analysis

 The study analyzed the data quantitatively using the Agreement Index (AI) to evaluate the results of the experts and the target audience, considering the answers classified as three and four (“I somewhat agree” and “I strongly agree”), divided by the total number of answers. According to this index, the items on content, face, motivation and usability that reached an agreement percentage among the participants equal to or greater than 80%, as recommended in the literature ^(26)^, were considered valid. 

 The Usability Score for the SUS scale was calculated by adding up the individual scores for each item. For odd-numbered items, one point is subtracted from the value given to that answer. For even items, the calculation is made by subtracting the value of the answer from the total of five points. The values obtained for odd and even items are added together and multiplied by 2.5 to obtain the total usability score, which varies between 0 and 100 points ^(23)^. It is worth noting that scores between 0 and 25 on the general Usability Score of the SUS scale indicate the worst achievable usability degree; from 26 to 39 is considered bad; from 40 to 52 means acceptable; from 53 to 74 is good; from 75 to 85 represents excellent; finally, from 86 to 100 is the best achievable ^(27)^. 

 Using the SUS scale, it is also possible to evaluate five key aspects of an app usability: 1) Ease of knowing the system - items 3, 4, 7 and 10; 2) System efficiency - items 5, 6 and 8; 3) Inconsistencies - item 6; 4) Ease of memorization - item 2; 5) User satisfaction - items 1, 4 and 9. The score of each participant’s answers to the items in each domain was multiplied by 25 to score the usability characteristics on a scale from 0 to 100. Subsequently, both in the validation and in the evaluation phase, the overall mean of the scores for each question was calculated by adding up the points awarded by all the participants and dividing by the total number of respondents. Afterwards, the mean of the items related to the specific characteristics of each domain was calculated. To do this, the points assigned to all the items in the domain were added up and divided by the total number of corresponding items ^(27 - 28)^. The items that did not reach the acceptable mark were revised. 

### Ethical aspects

 In order to comply with the ethical guidelines established by Resolution 466/2012 of the Brazilian National Health Council, this study was approved by the Research Ethics Committee (*Comitê de Ética em Pesquisa*, CEP) of *Universidade Federal Fluminense* (UFF) under opinion number 5,900,588 and CAAE 66077222.8.0000.5243. In addition, the participants were provided with an Informed Consent Form (ICF). 

## Results

 Stages 1, 2 and 3 resulted in the construction of the app called “*Nasci Bem*”. With various web and mobile specialization courses, the programming language used was Javascript in conjunction with the React Native Framework. The pastel blue and green colors of the booklet were kept in the interface. The illustrations for the booklet were obtained from Canva using a PRO account, with the exception of the logo, which was specifically created for this product. The tab icons were generated from Freepik images available on the Google platform. 

 When “*Nasci Bem*” is opened, an explanatory balloon about the technology appears in the center of the screen. After closing it, the logo is displayed with a prompt to choose between two access options: “*Profissional de saúde*” (“Health professional”) or “*Familiar de recém-nascido*” (“Family member of the newborn”). Three tabs appear when one of them is selected: “*Cartilha*” (“Booklet”), “Quiz” and “*Quem somos*” (“About us”). The “Booklet” tab content is adapted to the user’s profile. 

Both tabs offer similar content, but with language tailored to each audience: presentation; good practices; good vitality identification; what should be done immediately after delivery; practices to be postponed; vitamin K administration; neonatal ophthalmia prevention; examples of simple care to prevent avoidable deaths; support materials; and references.

The “Quiz” tab offers 10 questions for the users to assess their knowledge, clarifying possible doubts about the practices carried out with newborns in the maternity ward in an agile way, offering two answer options (True or False). After selecting an option, the correct answer is highlighted in a more vibrant shade: green for True and red for False. In addition, an explanatory text is displayed for each answer, highlighting the correct one and putting it into context.

**Figure 1:**
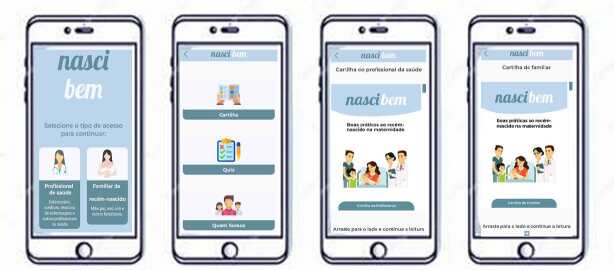
- Screenshots of the “*Nasci Bem*” app. Rio das Ostras, RJ, Brazil, 2023

 If the user chooses to exit the quiz before finishing all the questions, a message will appear: “*Retornar para o menu? Você perderá seu progresso*” (“Return to menu? You will lose your progress”). This will be followed by the “ *Ir para o menu*” (“Go to menu”) and “*Cancelar* ” (“Cancel”) options. After answering all the questions, the number of correct answers will be displayed. In addition, the “About us” section, available to both audiences, highlights the creators of the educational technology, the project that originated the app, its objectives and links to other technological products developed by the research team. The app validation and evaluation stages (4, 5, 6 and 7) are described below. 

The participants of the validation process (stage 4) were total of 22 experts, with a mean age of 42 years old (from 25 to 70). They were all female nurses, with four (18%) having a specialization degree, 12 (55%) a PhD and six (27%) an MSc. As for the specialization areas, six (27%) were in Pediatrics and Neonatology, five (23%) in Pediatrics, three (14%) in Neonatology, and one (4%) in each of the following areas: Children’s and Adolescents’ Health, Neonatal and Pediatric Intensive Care Unit, Neonatology and Perinatology, and Obstetrics. In relation to length of professional experience, 20 (91%) had been working for more than five years.

Table 1 shows the experts’ validation in terms of content and face, according to the AI values by item and overall. 

**Table 1 -:** Experts’ validation for the content and face of the first version of the app (n = 22). Rio das Ostras, RJ, Brazil, 2023

**Content**			
**Item**	**I strongly or somewhat disagree**	**I strongly or somewhat agree**	Item AI*
1) The app makes it easier to learn the concepts used and their applications.	0	22	1
2) The app is appealing to pregnant/puerperal women, family members of newborns and health professionals that work in maternity wards.	1	21	0.95
3) The app provides comprehensive help.	0	22	1
4) Information that promotes humanized care practices for newborns is covered.	0	22	1
5) It invites and/or instigates changes in the population (pregnant women, puerperal women, family members of newborns and health professionals working in maternity wards).	0	22	1
6) The app is suitable for use by anyone in the population.	4	18	0.82
7) The app content corresponds to the one found in the scientific literature.	0	22	1
8) The app is suitable for its intended purpose.	0	22	1
**Face**			
**Item**	**I strongly or somewhat disagree**	**I strongly or somewhat agree**	Item AI*
1) The letters are in an appropriate size.	3	19	0.86
2) The app interface is appealing.	1	21	0.95
3) The images are easy to understand.	0	22	1
4) The colors are appropriate.	0	22	1
5) The number of questions on the questionnaire is adequate.	0	22	1
6) The app looks organized.	0	22	1
7) All screens keep common app menus and functions accessible.	0	22	1
**Overall Agreement Index = 0.97**			

* AI = Agreement Index

The mean AI for all the items was above 0.8 (80%), both for content and face, showing that the app received a satisfactory evaluation, reaching an overall mean index of 0.97 (97%). The evaluation items ranged from 0.82 (82%) to 1.0 (100%).

In the evaluation (stage 6), 21 individuals from the target audience took part: eight health professionals from the delivery room and/or rooming-in unit (38%) and 13 family members of newborns (62%), including pregnant women, puerperal women and family members of newborns. Among the professionals, two were nurses (9.5%) and six were nursing technicians (28.6%), with experience from three months to 12 years. Of the family members, 11 were mothers (52.4%) and two were aunts (9.5%). Five had Higher Education (23.8%), seven had High School (33.3%) and one had Elementary School (4.8%). Their age range varied from 19 to 43 years old, with a mean of 31.

Table 2 below shows the target audience’s assessment of the content, face and motivation, using the AI values: by item and overall. 

**Table 2 -:** Evaluation by the target audience regarding the content, face and motivation of the second version of the app (n = 21). Rio das Ostras, RJ, Brazil, 2023

**Content**			
**Item**	**I strongly or somewhat disagree**	**I strongly or somewhat agree**	Item AI*
1) The language used in the app is easy to understand.	0	21	1
2) The information is clearly given.	0	21	1
3) The app facilitates learning about humanized care practices in the maternity ward.	0	21	1
4) Invites and/or attracts changes in the care provided to the newborn in the maternity ward.	0	21	1
5) The quiz clearly addresses the main recommended, non-recommended and recommended care practices with criteria.	0	21	1
6) The quiz is appealing.	0	21	1
**Face**			
**Item**	**I strongly or somewhat disagree**	**I strongly or somewhat agree**	Item AI*
1) The letters are in an appropriate size.	0	21	1
2) The app’s interface is attractive.	0	21	1
3) The images are easy to understand.	0	21	1
4) The colors are appropriate.	0	21	1
5) The app seems organized.	1	20	0.95
6) All screens keep common application menus and functions accessible.	0	21	1
**Motivation**			
**Item**	**I strongly or somewhat disagree**	**I strongly or somewhat agree**	Item AI*
1) The app provides help in a positive way	0	21	1
2) The app led you to think about humanized care practices.	0	21	1
3) The app motivated you to change your habits in terms of caring for your newborn in the maternity ward.	0	21	1
**Overall Agreement Index = 0.99**			

* AI = Agreement Index

The mean AI for all the content, face and motivation items was above 0.8 (80%), indicating that the app had a satisfactory evaluation, reaching an overall mean value of 0.99 (99%). The evaluation items ranged from 0.95 (95%) to 1.0 (100%).

Table 3 shows the experts’ validation and the target audience’s assessment of usability, according to the AI per item and the overall Usability Score of the SUS scale. 

In the usability validation process using the SUS scale by the experts on the app’s first version, the AI for all items was equal to or greater than 0.8 (80%), ranging from 0.82 (82%) to 1.0 (100%) and with a mean of 0.92 (92%), indicating a satisfactory evaluation. In addition, the overall Usability Score using the SUS scale was 93, varying from 77.5 to 100, demonstrating a better achievable usability degree.

**Table 3 -:** Experts’ validation of the first version (n = 22) and the target audience’s evaluation of the second version of the app (n = 21) in terms of usability employing the System Usability Scale. Rio das Ostras, RJ, Brazil, 2023

	**First version**			**Second version**		
**Item**	**I strongly or somewhat disagree / I neither agree nor disagree**	**I strongly or somewhat agree**	Item AI* among the experts	**I strongly or somewhat disagree / I neither agree nor disagree**	**I strongly or somewhat agree**	Item AI* by among the target population
1. I would use this app frequently	2	20	0.91	1	20	0.95
2. I found the app unnecessarily complex.	20	2	0.91	17	4	0.8
3. I found the app easy to use.	0	22	1	0	21	1
4. I think I would need technical support to be able to use this app.	18	4	0.82	20	1	0.9
5. I thought that the various functions of the app were well integrated.	1	21	0.95	0	21	1
6. I thought that there was plenty of inconsistencies in this app.	22	0	0.95	20	1	0.9
7. I would imagine that most people would learn how to use it.	1	21	0.95	1	20	0.95
8. I found the app too heavy to use.	21	1	0.86	21	0	1
9. I felt very confident using the app.	0	22	1	1	20	0.95
10. I had to learn a number of things before I could continue using the app.	20	2	0.86	20	1	0.9
**Overall Agreement Index among the experts = 0.92**						
**Usability Score among the experts = 93**						
**Overall Agreement Index among the target audience = 0.93**						
**Usability Score among the target audience = 94**						

* AI = Agreement Index

Despite the satisfactory evaluation, some of the experts’ suggestions were incorporated into the app improvement process, considering their feasibility. Those that were taken into account included: larger font in the booklets; adapted language in the quiz for professionals and family members; explanation of technical terms; adjustments to the wording; inclusion of references; and addition of data in “About us” and of content on some diseases and practices.

After the adjustments, the target audience evaluated the app’s second version, obtaining AI values for all items equal to or greater than 0.8 (80%), ranging from 0.8 (80%) to 1.0 (100%) and with a mean of 0.93 (93%), again indicating a satisfactory evaluation. In addition, the overall Usability Score using the SUS scale was 94, ranging from 60 to 100, again classifying it as having a better achievable usability level.

 The content, face, motivation and usability items of the “*Nasci Bem*” app received excellent evaluations from the experts and the target audience, showing improvements in the overall mean AI of the items and in the Usability Score between the first and second version. 

The suggestions from the target audience included availability of the app in app stores, inclusion of information on vitamin K oral administration in the event of intramuscular administration being refused, and improvements to the distribution of the booklet content. After the final adjustments, the final version of the app will be released in app stores.

 Finally, the SUS scale elements have specific usability attributes that have relevant meanings, used to assess the quality aspects of software (Table 4). 

**Table 4 -:** Experts’ validation for the first version and the target audience’s assessment for the usability features of the second version (n = 43). Rio das Ostras, RJ, Brazil, 2023

	**First version**		**Second version**		**Meaning**
**Usability features**	**Mean of the items among the experts**	**Overall mean**	**Mean of the items among the target audience**	**Overall mean**	
Easy-to-understand system	I* 3 (100.0)	96.6	I* 3 (100.0)	94.6	Easy-to-use system when used for the first time
	I* 4 (100.0)		I* 4 (94.0)		
	I* 7 (96.6)		I* 7 (94.0)		
	I* 10 (89.8)		I* 10 (90.5)		
System efficiency	I* 5 (97.7)	93.2	I* 5 (96.4)	94.8	Speed in executing established tasks
	I* 6 (95.5)		I* 6 (90.5)		
	I* 8 (86.4)		I* 8 (97.6)		
Inconsistencies	I* 6 (95.5)	95.5	I* 6 (90.5)	90.5	No errors
Ease of memorization	I* 2 (92.0)	92.0	I* 2 (86.9)	86.9	Easy-to-use system even after a long period without using it
User satisfaction	I* 1 (89.8)	95.8	I* 1 (94.0)	94.4	Nice design
	I* 4 (100.0)		I* 4 (94.0)		
	I* 9 (97.7)		I* 9 (95.2)		

* I = Item

The results showed that, from the first version onwards, all the items obtained scores higher than 86 in the app’s usability features. This indicates that all of them achieved scores classified as the best achievable, making it an educational technology with high ease of knowledge and system memorization, as well as high satisfaction and efficiency, and low inconsistency.

## Discussion

The study satisfactorily achieved its objective of constructing, validating, and evaluating a mobile app on humanized care practices for newborns at habitual risk in maternity wards. The app is aimed at health professionals, pregnant/puerperal women and family members of newborns, as the agreement index achieved on content, face, motivation and usability (both by item and overall) was above the desirable values. In addition, from the first version onwards, usability reached a level classified as “the best achievable”, indicating excellent potential for use by the target audience.

 It is important to emphasize the importance of creating educational technologies in health, as well of their validation by experts and evaluation with the target audience, especially those related to topics of interest to the population and aligned with their learning needs ^(29)^. Through these technological products it is possible to increase access to quality health information, fostering autonomy and empowerment of both health professionals and families in promoting good care practices for habitual-risk newborns in maternity wards. 

 Based on the scientific evidence, there has been a global development of educational technologies in the form of mobile apps, with the aim of instructing, informing and guiding. One study created and evaluated a Serious Game focused on improving the care of premature newborns by their families, obtaining results that converge with the current study. The game’s evaluation revealed satisfactory content, face and usability levels, in addition to stimulating the participants in the learning process ^(29)^. 

 Another study developed, validated and evaluated a breast milk milking technology for nurses working in agribusinesses. This research validated the Content Validity Index (CVI) with experts at 86.72% and evaluated the game’s usability in app form by these nurses, obtaining a score of 83.89 ^(30)^. These scores were lower than those presented in this study, which used the same evaluation scale. Another study developed and validated a mobile app for pregnant women undergoing prenatal care, covering questions about breastfeeding. This study achieved a CVI of 0.89 among the experts, showing reliability of the information and the technical side of the system. This app has potential as a resource for promoting health during the pregnancy and puerperal periods ^(13)^. 

 One project created the “*Preemie Care*” app to guide neonatal assistance by health professionals, evaluating its usability with specialists using the SUS scale and achieving a score of 88%. This indicated acceptable and excellent overall usability ^(18)^, coming close to the current study, which obtained “the best achievable” rating in all the system features. 

 In the process of constructing, validating and evaluating the “ *Descomplicando a amamentação*” (“Uncomplicating Breastfeeding”) app, the validation and evaluation stages were conducted in separate studies. In the content, face and usability validation using the CVI, a mean score of 0.96 ^(22)^was obtained. In the semantics, face and usability evaluation using the AI and the SUS scale, respectively, values of 0.99 and 93 were achieved, indicating satisfactory and highly effective results for use by families ^(31)^. These results are similar to those obtained in this study, which were superior in some aspects. 

 Only one of the surveys that were found performed validation and evaluation procedures in the same study ^(30)^. Although all the items achieved excellent results, the current survey outperformed both the validation with experts and the evaluation with the target audience. It is worth noting that most localized mobile apps aimed at this population focus on high-risk pregnancies and on the specific care of premature newborns. However, what sets this study apart is its approach to normal-risk pregnancies and newborns, thus making a significant contribution to maternal and children’s health. 

 International studies have shown that using mobile apps about pregnancy, labor and birth is effective in improving health professionals’ and family members’ knowledge, providing information and empowerment to the users ^(32 - 33)^. They can also support communication between families and professionals during the prenatal period, strengthening decision-making ^(34)^ and, thus, reinforcing the importance of the current study. 

 For these technologies to be effective, it is fundamental to consider essential aspects that determine the software’s quality, as assessed by the SUS scale in this study. This instrument covers various aspects of the system, such as interface, necessary support and complexity, among others. Therefore, this evaluation tool has high validity in measuring usability of an app, making it a reliable and robust evaluation tool ^(27)^. 

 This classification assessed the “*Nasci Bem*” mobile app as a device with the best achievable usability, portraying ease of use, satisfaction, efficiency and low inconsistency perceived by the users. Usability testing is therefore essential before making an app available to the public, as it is one of the main criteria for ensuring that a mobile app is easy for users to use and achieves its proposed objectives ^(35)^. 

The study limitations include selection by convenience, restricting the analysis to the participants selected, as well as dependence on access to the Internet and mobile devices, highlighting the need for further studies with different audiences and other methodological approaches.

 Availability of the “*Nasci Bem*” app can reduce inappropriate practices such as separation of mother and newborn at birth, early clamping of the umbilical cord and aspiration of the airway and gastric tract in the delivery room, as well as adoption of appropriate practices like early breastfeeding and skin-to-skin contact. The technology gathers humanized care practices for newborns, covering up-to-date recommendations in a single place and empowering the target audience, who can access this information any time, promoting greater autonomy. The aim is to reduce neonatal and infant morbidity and mortality and to expand digital health. 

## Conclusion

 The health professionals and family members that took part in the methodological stages of this study considered the “*Nasci Bem*” app to be understandable, relevant, pertinent, easy to use, with low inconsistency and with excellent potential for use as an educational technology in health. 

This innovative tool emerges as a facilitating instrument for health professionals, providing training in humanized practices for newborns. It also offers support to pregnant/puerperal women and family members, allowing them to build knowledge from prenatal care onwards about the best newborn care practices, making learning practical and accessible.

It is available for free download for the Android operating system, on the Google Play Store platform, and will soon also be available for iOS, on the Apple Store.

## References

[B1] Schott L. C., Góes F. G. B., Santos A. S. T., Silva A. C. S. S., Pereira-Ávila F. M. V., Goulart M. C. L. (2022). Adherence to humanized care practices for newborns with good
vitality in the delivery room. Rev Gaúcha Enferm.

[B2] World Health Organization (2018). WHO recommendations: intrapartum care for a positive childbirth
experience [Internet].

[B3] Ledo B. C., Góes F. G. B., Santos A. S. T., Pereira-Ávila F. M. V., Silva A. C. S. S., Bastos M. P. C. (2021). Factors associated with care practices for newborns in the
delivery room. Esc Anna Nery.

[B4] Carmo M. M., Lima E. S. (2022). Good practices in nursing care for healthy
newborns. Braz J Develop.

[B5] Silva J. L. P., Linhares F. M. P., Barros A. A., Souza A. G., Alves D. S., Andrade P. O. N. (2018). Factors associated with breastfeeding in the first hour of life
in a baby-friendly hospital. Texto Contexto Enferm.

[B6] Jardim T. S., Viana G. P., Cruz W. O., Assis T. O., Lemos G. D., Almeida K. J. S. (2019). Principais fatores relacionados à impossibilidade de amamentação
em puérperas assistidas no Isea. Braz J Health Rev [Internet].

[B7] Silva M. F. F. S., Pereira L. B., Ferreira T. N., Souza A. A. M. (2018). Breastfeeding self-efficacy and interrelated
factors. Rev Rene.

[B8] Gonçalves M. O. S. S., Silva M. L., Silva J. D. A., Roxa G. N., Tavares M. J. A., Pedro U. N. S. F. (2021). Maternal factors related to cesarean indication: an integrative
literature review. Braz J Develop.

[B9] Dias B. A. S., Leal M. C., Esteves-Pereira A. P., Nakamura-Pereira M. (2022). Variations in cesarean and repeated cesarean section rates in
Brazil according to gestational age at birth and type of
hospital. Cad Saúde Pública.

[B10] Ayres L. F. A., Cnossen R. E., Passos C. M., Lima V. D., Prado M. R. M. C., Beirigo B. A. (2021). Factors associated with early skin-to-skin contact in a maternity
hospital. Esc Anna Nery.

[B11] Holztrattner J. S., Gouveia H. G., Moraes M. G., Carlotto F. D., Klein B. E., Coelho D. F. (2021). Early skin-to-skin contact in a child friendly hospital:
perceptions of the obstetric nurses. Rev Gaúcha Enferm.

[B12] Góes F. G. B., Silva M. A., Santos A. S. T., Pontes B. F., Lucchese I., Silva M. T. (2020). Postnatal care of newborns in the family context: an integrative
review. Rev Bras Enferm.

[B13] Souza F. M., Santos W. N., Dantas J. C., Sousa H. R., Moreira O. A., Silva R. A. (2022). Development of a mobile application for prenatal care and content
validation. Acta Paul Enferm.

[B14] Meirelles F. S. (2022). Tecnologias de informação: 33° pesquisa anual do uso de TI
[Internet].

[B15] Abernethy A., Adams L., Barrett M., Bechtel C., Brennan P., Butte A. (2022). The promise of digital health: then, now, and the future
[Internet]. National Academy of Medicine.

[B16] Silva A. M. A., Mascarenhas V. H. A., Araújo S. N. M., Machado R. S., Santos A. M. R., Andrade E. M. L. R. (2018). Mobile technologies in the nursing area. Rev Bras Enferm.

[B17] Ministério da Saúde (BR) (2020). Secretaria-Executiva, Departamento de Informática do SUS. Estratégia de
Saúde Digital para o Brasil 2020-2028 [Internet].

[B18] Gaspar J., Vera-Montoya M. A., Lage E. M., Penido M. G., Ferreira R. L., Ramos I. J. (2022). Usability assessment of an app to guide neonatal care: the
preemie care. Rev Saúde Digital Tec Educ [Internet].

[B19] Porto A. F. (2022). Desenvolvimento de website para gestantes: estudo metodológico
[Thesis].

[B20] Franco G. A. S. (2021). Aplicativo móvel para orientações de familiares de crianças e
adolescentes em tratamento com quimioterapia antineoplásica oral
[Thesis].

[B21] Teixeira E., Mota V. M. S. S. (2011). Tecnologias educacionais em foco.

[B22] Souza N. A., Góes F. G. B., Mello N. C., Silva L. F., Silva A. C. S. S., Barcellos T. M. T. (2021). Educational technology about breastfeeding for mobile
devices. Cogitare Enferm.

[B23] Brooke J. (1993). Chapter 21, SUS: A “Quick and Dirty” usability scale.

[B24] Tenório J. M., Cohrs F. M., Sdepanian V. L., Pisa I. T., Marin H. F. (2010). Desenvolvimento e avaliação de um protocolo eletrônico para
atendimento e monitoramento do paciente com doença celíaca. Rev Inform Teor Apl.

[B25] Pereira F. G. F., Rocha D. J. L., Melo G. A. A., Jaques R. M. P. L., Formiga L. M. F. (2019). Building and validating a digital application for the teaching of
surgical instrumentation. Cogitare Enferm.

[B26] Miranda F. D., Salomé G. M. (2022). Development of a mobile app to assess, treat and prevent pressure
injury. Acta Paul Enferm.

[B27] Cavalcanti H. G. O., Bushatsky M., Barros M. B. S. C., Melo C. M. C. S., Delgado  A. J. F. D. (2021). Evaluation of the usability of a mobile application in early
detection of pediatric cancer. Rev Gaúcha Enferm.

[B28] Melo C. M. C. S., Delgado  A. J. F., Oliveira E. R., Araújo A. A., Cavalcanti H. G. O., Melo C. M. C. S. (2020). Development and assessment of an application for primary care for
users with diabetes mellitus. Aquichan.

[B29] D’Agostini M. M., Aredes N. D. A., Campbell S. H., Fonseca L. M. M. (2020). Serious Game e-Baby Família: an educational technology for
premature infant care. Rev Bras Enferm.

[B30] Moraes V. C., Ferraz L. (2021). Educational technology on expressing breast milk: development and
validation of a Serious Game. Rev Bras Saúde Mater Infant.

[B31] Lucchese I., Góes F. G. B., Souza A. N., Silva A. C. S. S., Silva L. F., Soares I. A. A. (2023). Evaluation of the mobile application "Descomplicando a
Amamentação" by family members of newborns. Rev. Latino-Am. Enfermagem.

[B32] Shimpuku Y., Mwilike B., Mwakawanga D., Ito K., Hirose N., Kubota K. (2023). Development and pilot test of a smartphone app for midwifery care
in Tanzania: A comparative cross-sectional study. PLoS One.

[B33] Mueller S., Soriano D., Boscor A., Saville N. M., Arjyal A., Baral S. (2020). MANTRA: improving knowledge of maternal health, neonatal health,
and geohazards in women in rural Nepal using a mobile serious
game. Front Public Health.

[B34] Bailey E., Nightingale S., Thomas N., Coleby D., Deave T., Goodenough T. (2022). first-time mothers’ understanding and use of a pregnancy and
parenting mobile app (The Baby Buddy App): qualitative study using
appreciative inquiry. JMIR Mhealth Uhealth.

[B35] Marques A. D. B., Moreira T. M. M., Jorge T. V., Rabelo S. M. S., Carvalho R. E. F. L., Felipe G. F. (2020). Usability of a mobile application on diabetic foot
self-care. Rev Bras Enferm.

