# Supplementary material for: The “Nasci Bem” app for health professionals and families of newborns: construction, validation and evaluation
Source: Rev Lat Am Enfermagem. 2024 Nov 22;32:e4260. doi: 10.1590/1518-8345.7264.4260 (PMC11654027; doi:10.1590/1518-8345.7264.4260)
Supplement: Translation

# Translation: Aplicación “*Nasci Bem*” para profesionales de la salud y familiares de recién nacidos: creación, validación y evaluación *

**DOI:** 10.1590/1518-8345.7264.4259

**Published:** 2024-11-15

**Authors:** Ingrid Lucchese, Fernanda Garcia Bezerra Góes, Maithê de Carvalho e Lemos Goulart, Maria da Anunciação Silva, Aline Cerqueira Santos Santana da Silva, Liliane Faria da Silva

**Affiliations:** ^1^ Universidade Federal Fluminense, Instituto de Humanidades e Saúde, Rio das Ostras, RJ, Brasil.; ^2^ Becaria del Conselho Nacional de Desenvolvimento Científico e Tecnológico (CNPq), Brasil.; ^3^ Universidade Federal do Estado do Rio de Janeiro, Rio de Janeiro, RJ, Brasil.; ^4^ Universidade Federal Fluminense, Escola de Enfermagem Aurora de Afonso Costa, Niterói, RJ, Brasil.

**Keywords:** Aplicaciones Móviles, Alojamiento Conjunto, Maternidades, Recién Nacido, Salas de Parto, Tecnología Educacional

## Abstract

crear, validar y evaluar una aplicación para dispositivos móviles sobre prácticas de atención humanizada al recién nacido de riesgo habitual en la maternidad, dirigida a profesionales de la salud y familiares.

estudio metodológico con siete etapas: revisión de la literatura; organización del contenido; creación de la aplicación; validación por expertos; adecuación después de la validación; evaluación por parte del público objetivo; y adecuación después de la evaluación. En la evaluación participaron 22 expertos en validación y 21 personas del público objetivo. Los instrumentos abordaron contenido, apariencia y/o motivación, con la *System Usability Scale* para usabilidad. Se calcularon: Índice de Concordancia y Puntaje de Usabilidad.

en la validación, la aplicación alcanzó un Índice de Concordancia de 0,97; osciló entre 0,82 y 1,0 en los ítems analizados. En la evaluación el índice fue de 0,99; varió de 0,95 a 1,0. En la usabilidad de la primera versión, el Índice de Concordancia fue de 0,92 y el puntaje de usabilidad fue de 93. En la segunda, el índice fue de 0,93 y un puntaje de 94, lo que indica que la usabilidad era la mejor posible desde la primera versión.

la aplicación “*Nasci Bem*” demostró que era comprensible, relevante, pertinente, eficiente, fácil de usar y de baja inconsistencia y que tenía un excelente potencial de uso como tecnología educativa para los profesionales y las familias.

## Destacados

**(1)** El estudio creó, validó y evaluó la aplicación móvil “ *Nasci Bem*”.

**(2)** Participaron de la investigación 22 expertos y 21 profesionales de la salud y familiares.

**(3)** La validación y la evaluación presentaron altos niveles de concordancia y usabilidad.

**(4)** La tecnología educativa es comprensible, relevante, eficiente y fácil de usar.

**(5)** La aplicación “Nasci Bem” contribuye a la salud digital.

## Introducción

 El Programa Brasileño de Humanización del Prenatal y del Nacimiento tiene como objetivo mejorar la atención prenatal y la atención al parto y posparto de la madre y el bebé, al promover un abordaje humanizado del parto como una experiencia verdaderamente humana ^(1)^. Según las directrices globales de la atención a gestantes sanas de la Organización Mundial de la Salud (OMS), no se recomienda la intervención inmediata del equipo de salud en situaciones de riesgo habitual, y enfatiza que hay que respetar la particularidad de cada mujer y su proceso de parto para que tenga una experiencia positiva. Es fundamental hacerlas partícipe de las decisiones sobre la atención que reciben, incluso cuando es necesario que intervengan los profesionales ^(2)^. 

 En Brasil, aún predomina el modelo tecnocrático de atención durante el parto, que se centra en los profesionales de la salud y es propenso a intervenciones inadecuadas ^(3)^. Por ende, no se están adoptando de manera efectiva las prácticas humanizadas que recomienda la OMS, como el contacto temprano piel con piel, que fomenta la lactancia materna, previene la hipotermia neonatal y fortalece el vínculo entre la madre y el bebé; la lactancia materna en la primera hora de vida, que reduce el destete precoz y favorece la salud cardiorrespiratoria; el pinzamiento oportuno del cordón umbilical, que reduce la anemia en los primeros meses de vida; y la presencia de un acompañante en la sala de partos, que se sabe que promueve las buenas prácticas ^(1 - 3)^. 

 Es necesario sensibilizar a los profesionales de la salud sobre las prácticas humanizadas en las salas de maternidad, que brindan comodidad y seguridad y disminuyen las cesáreas innecesarias ^(4)^, que dificultan, pero no impiden, el contacto piel con piel y la lactancia precoz, debido a la anestesia y posición de la cama a cero grados ^(5)^. Además, las mujeres sometidas a una cesárea liberan menos oxitocina inmediatamente después del nacimiento, una hormona crucial para que tengan un buen flujo de leche materna ^(6)^, y sienten más dolor y malestar posquirúrgico ^(7)^. Sin embargo, la tasa de cesáreas en Brasil está alrededor del 55% ^(9)^, supera ampliamente los índices recomendados por la OMS que van entre el 10% y 15% ^(8)^, y limita la adopción efectiva de prácticas humanizadas. Por otro lado, las prácticas ideales para el cuidado del bebé después de una cesárea deben ser las mismas que para el parto vaginal ^(4)^.

 Varios factores favorecen la adherencia a prácticas humanizadas en recién nacidos de riesgo normal en la maternidad, especialmente el parto vaginal, la buena vitalidad y el contacto piel con piel temprano, que facilita el pinzamiento oportuno del cordón umbilical y la lactancia materna en la primera hora de vida ^(1 - 3)^. Si bien algunos recién nacidos requieren intervenciones, en muchos centros hospitalarios, aún se realizan prácticas en recién nacidos sanos ^(3)^ como la separación binomial, el uso de oxígeno inhalado, la aspiración oronasofaríngea, gástrica y traqueal ^(1, 10)^ y el pinzamiento temprano o inmediato del cordón umbilical ^(4)^. En ocasiones, se eligen esas intervenciones, incluso sin condiciones patológicas que las justifiquen ^(10)^.

 Los profesionales de la salud han demostrado que tienen conocimientos sobre las prácticas humanizadas, lo que se considera un punto de partida fundamental para implementarlas, según la literatura ^(11)^. Pero la adhesión efectiva requiere concientización por parte de los gestores y profesionales, apoyo institucional y actualización basada en evidencia. Además, se recomienda difundir orientaciones sobre una atención posnatal segura, desde la atención prenatal hasta la transición de la maternidad al hogar ^(12)^, para capacitar a las gestantes, las puérperas y los familiares. 

 Las tecnologías educativas en salud, integradas a la vida cotidiana de los pacientes y profesionales, tienen el potencial de promover la salud a través de información sobre la prevención y el tratamiento de problemas de salud ^(13)^. Según la 33ª Encuesta Anual sobre el Uso de Tecnologías de la Información de la Fundación Getúlio Vargas, en 2022, Brasil tenía 447 millones de dispositivos digitales (computadoras, *notebooks*, *tablets* y *smartphones*), más de dos por habitante ^(14)^. En virtud de la presencia generalizada de estos dispositivos, especialmente de *smartphones*, el uso de tecnologías educativas es un recurso promisorio para que los enfermeros actúen de forma dinámica como educadores en salud. A nivel mundial, la salud digital es reconocida como una herramienta eficaz para promover la información en salud, que llega a diferentes públicos ^(15 - 16)^.

 Brasil, en 2020, adhiriendo a las recomendaciones mundiales, lanzó el Plan de Acción de Salud Digital 2020-2028, que describe estrategias para lograr la Visión de Salud Digital. Este plan propuso la creación de una plataforma integrada para promover el intercambio de datos en la Red de Atención a la Salud, con la expectativa de llegar a implementarlo en las 27 unidades federativas hasta finales de 2023 ^(17)^. Este desarrollo apoya el diseño de herramientas digitales de salud orientadas a la educación para la salud, que faciliten la atención continua, independientemente del momento o lugar. 

 En la literatura se identificaron investigaciones que crearon aplicaciones en el área, una para gestantes en control prenatal, con el objetivo de promover la salud durante el período embarazo-puerperio ^(13)^, y otra dirigida a los profesionales de la salud para evaluar riesgos para la salud neonatal y difundir prácticas para la atención al recién nacido ^(18)^. Sin embargo, se observó que hay lagunas de conocimiento en la literatura, debido a la escasez de estudios internacionales y nacionales sobre aplicaciones que aborden prácticas de atención humanizada al recién nacido de riesgo habitual en las maternidades, dirigidas a los profesionales y familiares simultáneamente. Así surgió la intención de crear una aplicación móvil para ello, una tecnología educativa que contribuya a que los profesionales de la salud tomen decisiones asertivas y que les brinde conocimientos prácticos y accesibles a los familiares desde la atención prenatal.

Esta investigación tiene como objetivo contribuir al avance del conocimiento científico y la salud digital, al presentar el proceso completo desde la concepción hasta la evaluación de una aplicación innovadora que reúne contenidos actualizados sobre las prácticas de atención al recién nacido: recomendadas, recomendadas con criterio y no recomendadas, en forma móvil, accesible y de libre acceso, lo que refuerza la relevancia de este estudio.

Por lo tanto, el objetivo del estudio fue crear, validar y evaluar una aplicación para dispositivos móviles sobre prácticas de atención humanizada al recién nacido de riesgo habitual en la maternidad dirigida a profesionales de la salud y familiares.

## Método

### Tipo de estudio

 Estudio metodológico con enfoque cuantitativo que creó, validó y evaluó una tecnología educativa en forma de aplicación móvil en siete etapas: 1) Revisión de la literatura; 2) Organización del contenido; 3) Creación de la aplicación (diseño y desarrollo); 4) Validación de la aplicación por expertos; 5) Adecuación después de la validación; 6) Evaluación de la aplicación por parte del público objetivo; y 7) Adecuación después de la evaluación. Los estudios metodológicos generalmente son no experimentales y se centran en la creación de nuevos materiales educativos ^(19 - 20)^.

### Escenario

El estudio se desarrolló en un entorno virtual.

### Período

El desarrollo de la aplicación comenzó en enero de 2023 y la recopilación de datos para su validación y evaluación se llevó a cabo entre agosto y noviembre de 2023.

### Población

La población de estudio estuvo compuesta por expertos (profesionales de la salud con experiencia en neonatología, pediatría u obstetricia) y público objetivo (profesionales de la salud de maternidades y gestantes, puérperas y familiares de recién nacidos).

### Criterios de selección

 Para seleccionar a los expertos se utilizaron los siguientes criterios de inclusión: profesionales de la salud con experiencia en neonatología, pediatría u obstetricia; y se aplicó el criterio de Fehring ^(20)^, que asigna un puntaje de 1 a 5 puntos y considera: participación en eventos científicos en los últimos dos años sobre el tema (1 punto), experiencia mínima de cinco años en el área (2 puntos), publicación en revistas indexadas sobre el tema (2 puntos), título de especialista (3 puntos), título de maestría (4 puntos) y título de doctorado (5 puntos). Para calcular el puntaje se consultaron los datos presentes en el CV de la Plataforma Lattes de cada profesional seleccionado. Se excluyó a quienes no alcanzaron un puntaje mínimo de cinco puntos. Otro criterio de exclusión fue profesionales que desempeñaban solamente actividades administrativas. 

 El público objetivo se seleccionó en base a los criterios de inclusión: profesionales de la salud que trabajan en sala de parto y/o alojamiento conjunto, gestantes, puérperas y familiares de recién nacidos mayores de 18 años, con acceso a internet. Los criterios de exclusión fueron profesionales de la salud que desempeñaban funciones como auxiliares de enfermería, no brindaban atención directa al binomio mamá-bebé o se dedicaban solamente a actividades administrativas. Además, gestantes, mujeres que hayan dado a luz recientemente o familiares de recién nacidos con limitaciones físicas y/o mentales para responder formularios *online* o que sean analfabetos. 

### Definición de la muestra

 Los participantes fueron invitados mediante selección por conveniencia y no probabilística. La muestra estuvo compuesta por 22 expertos y 21 personas del público objetivo, que entregaron sus evaluaciones en el plazo estipulado de diez días. Esta distribución sigue la recomendación de la literatura sobre el desarrollo de tecnologías educativas, que indica que debe haber al menos nueve participantes en cada grupo de evaluadores ^(21)^. El grupo correspondiente al público objetivo estuvo conformado por 8 profesionales de la salud y 13 gestantes, puérperas y familiares de recién nacidos. 

### Variables del estudio

La primera sección del instrumento de validación para los expertos incluyó preguntas cerradas para caracterizar a los participantes, como edad, género, formación y área de actividad profesional, cualificaciones y tiempo de experiencia. En el instrumento de valoración del público objetivo se incluyeron aspectos como edad, sexo, nivel educativo, profesión, tipo de relación (profesional de la salud o familiar de un recién nacido), tiempo de trabajo en la maternidad (para profesionales de la salud) y vínculo con el recién nacido (para miembros de la familia). La segunda sección abordó temas específicos relacionados con el análisis de la tecnología educativa.

### Instrumentos utilizados para recopilar información

 El proceso de recolección de datos para la validación y evaluación de la aplicación móvil se realizó mediante *Google Forms* en la plataforma *Google*. La página de la encuesta *online* contenía información detallada sobre el proyecto, el Registro de Consentimiento Libre e Informado (RCLI) disponible para que los participantes lo descarguen y el formulario electrónico para la recolección de datos. 

 El instrumento de validación de la aplicación para los expertos, previamente validado, incluyó ocho preguntas sobre contenido y siete sobre apariencia. Para la evaluación por parte del público objetivo se utilizó un instrumento, también validado previamente, con seis preguntas sobre contenido, seis sobre apariencia y tres sobre motivación. Para evaluar los ítems se usó una escala Likert de 1 a 4 puntos, a saber: “Estoy muy en desacuerdo” (1 punto), “Estoy poco en desacuerdo” (2 puntos), “Estoy poco de acuerdo” (3 puntos) y “Estoy muy de acuerdo” (4 puntos) ^(22)^. 

 Secuencialmente, ambos grupos de evaluadores utilizaron la *System Usability Scale* (SUS) ^(23)^, compuesta por diez ítems. Esta escala utiliza un puntaje que va de 1 a 5 puntos, que representan, respectivamente: “Estoy muy en desacuerdo” (1 punto), “Estoy un poco en desacuerdo” (2 puntos), “No estoy ni de acuerdo ni en desacuerdo” (3 puntos), “Estoy un poco de acuerdo” (4 puntos) y “Estoy muy de acuerdo” (5 puntos) ^(24)^. Además, al final de los instrumentos se asignó un espacio para sugerencias y comentarios para mejorar la tecnología educativa en salud. 

### Recolección de datos

 La primera etapa, revisión de la literatura del tipo narrativa, tuvo como objetivo identificar, recopilar y sintetizar información sobre las prácticas de atención recomendadas, recomendadas con criterio y no recomendadas para la atención del recién nacidos de riesgo habitual en la maternidad. Basándose en autores especialistas en el área, documentos del Fondo de las Naciones Unidas para la Infancia (UNICEF), la OMS, el Ministerio de Salud (MS) de Brasil y la Sociedad Brasileña de Pediatría (SBP), la revisión buscó publicaciones de los últimos diez años, e incluyó artículos, manuales, guías, directrices y resoluciones relacionados con el tema de estudio. Esta búsqueda se realizó consultando directamente los sitios *web* de dichas organizaciones, para garantizar la confiabilidad y actualidad de la información recolectada, e incluyó términos como “Recién Nacido”, “Maternidad”, “Salas de Parto” y “Alojamiento Conjunto”. 

 En la segunda etapa, correspondiente a la organización del contenido, utilizamos la evidencia científica encontrada en la literatura para crear una matriz de análisis con los principales hallazgos en los sitios *web* de las organizaciones científicas mencionadas. Una vez que se identificaron las prácticas, se extrajeron las conductas esenciales que había que implementar en la sala de parto y en el alojamiento conjunto, para promover prácticas de atención humanizadas. A partir de esta organización, el equipo desarrolló el contenido para la tecnología, que incluyó textos, imágenes y *links*. El objetivo fue crear una guía para que la creación de la aplicación se realice de manera creativa, atractiva, intuitiva, detallada, motivadora y efectiva. 

 En la tercera etapa, referente a la creación de la aplicación móvil, se realizó la primera reunión entre el equipo de estudio y un desarrollador especializado en Informática, quien tiene una empresa de servicios en la zona. Cabe destacar que el desarrollo de la aplicación, paso decisivo para la implementación, debe ser realizado por profesionales especialistas, con la supervisión de investigadores, como lo indica otro estudio ^(25)^. Durante la reunión inicial, se discutieron ideas para el diseño instruccional y la estructura de navegación, adaptadas en forma de una aplicación de descarga gratuita. La interfaz fue diseñada para garantizar interactividad, autonomía y accesibilidad en todos los comandos. 

 En un intervalo de 23 días se llevó a cabo una segunda reunión, en la que el desarrollador presentó una versión preliminar de la aplicación en formato *Android Application Pack* (APK). Esta versión ya tenía las pestañas con el material educativo, además de los colores y las fuentes predeterminadas, que permitían realizar ajustes según las preferencias del equipo. Posteriormente, se realizó una nueva etapa de inserción de datos en la herramienta educativa, que derivó en la creación de las interfaces: menú principal, folletos, cuestionarios e información sobre cada perfil de usuario, lo que dio origen a la primera versión de la aplicación. 

 En las etapas cuatro y seis, la recolección de datos se realizó a través de invitaciones por medio de *WhatsApp*, correos electrónicos e *Instagram*, en las que se invitaba a los participantes a validar o evaluar la aplicación móvil. Para preservar la privacidad, no se utilizaron listas de contactos identificables. La invitación incluía una breve explicación del proyecto, la identificación de las investigadoras, un *link* a la investigación y la aplicación en formato APK para descarga en *smartphones Android*. Se estableció un plazo de 10 días para enviar la respuesta, y un promedio de treinta minutos para analizar la aplicación y completar el formulario. 

Debido a que la muestra fue no probabilística, de tipo consecutivo, conformada durante el período previsto por los investigadores, la recolección de datos se llevó a cabo sin dificultades, y la mayoría de los expertos invitados y de las personas del público objetivo reenvió el material rápidamente, sin negativas ni demoras. Cabe señalar que, debido a que la comunicación se realizó a través de las redes sociales, algunas de las invitaciones enviadas no fueron respondidas, por eso, se distribuyeron más invitaciones de las que determinaba el límite mínimo establecido, aproximadamente 25 para expertos y 30 para el público objetivo.

Cabe destacar que la validación de la primera versión de la aplicación fue realizada por los expertos en la cuarta etapa. Sólo después de que se realizaran las adaptaciones sugeridas en la etapa cinco, se llevó a cabo la evaluación de la segunda versión con el público objetivo en la etapa seis. Sin embargo, después de haber completado esas fases, fue necesario realizar pequeñas modificaciones para obtener la versión final de la aplicación, que se completó en la séptima y última etapa.

### Tratamiento y análisis de los datos

 El análisis de los datos de realizó de forma cuantitativa, se utilizó el Índice de Concordancia (IC) para evaluar los resultados de los expertos y del público objetivo, se consideraron las respuestas con puntaje tres y cuatro (estoy poco de acuerdo y estoy muy de acuerdo) y se las dividió por el total de respuestas. Según este índice, se consideraron válidos los ítems sobre contenido, apariencia, motivación y usabilidad, que alcanzaron un porcentaje de concordancia, según los participantes, igual o superior al 80%, tal como recomienda la literatura ^(26)^.

 El cálculo del Puntaje de Usabilidad de la escala SUS se generó sumando los puntajes individuales de cada ítem. Para los ítems impares, se resta un punto del valor dado a esa respuesta. Cuando los ítems son pares, el cálculo se realiza restándole el valor de la respuesta al total de cinco puntos. Los valores obtenidos para los ítems pares e impares se suman y multiplican por 2,5 para obtener el puntaje total de usabilidad, que varía entre 0 y 100 puntos ^(23)^. Cabe mencionar que un puntaje entre 0 y 25 en el Puntaje de Usabilidad general de la escala SUS indica el peor grado de usabilidad posible; de 26 a 39 se considera malo; de 40 a 52 es aceptable; de 53 a 74 es bueno; de 75 a 85 es excelente y de 86 a 100 es el mejor posible ^(27)^.

 Utilizando la escala SUS, también se puede evaluar cinco aspectos claves para la usabilidad de una aplicación: 1) Facilidad de conocimiento del sistema - ítems 3, 4, 7 y 10; 2) Eficiencia del sistema - ítems 5, 6 y 8; 3) Inconsistencias - ítem 6; 4) Facilidad de memorización - ítem 2; y 5) Satisfacción del usuario - ítems 1, 4 y 9. El puntaje de las respuestas de cada participante a los ítems en cada dominio se multiplicó por 25 para calificar las características de usabilidad en una escala de 0 a 100. Luego, tanto en la fase de validación como en la de evaluación se calculó el promedio general de los puntuajes de cada pregunta, sumando los puntos otorgados por todos los participantes y dividiéndolos por el número total de encuestados. Posteriormente se calculó el promedio de los ítems relacionados con las características específicas de cada dominio. Para ello, se sumaron los puntos asignados a todos los ítems del dominio y se los dividió por el número total de ítems correspondientes ^(27 - 28)^. Los ítems que no alcanzaron una calificación aceptable requieren revisión.

### Aspectos éticos

 Para cumplir con las directrices éticas establecidas por la Resolución n.º 466/2012 del Consejo Nacional de Salud, este estudio fue aprobado por el Comité de Ética en Investigación (*Comitê de Ética em Pesquisa*, CEP) de la Universidad Federal Fluminense (UFF) con dictamen número 5.900.588 y CAAE: 66077222,8.0000.5243. Además, los participantes recibieron el RCLI. 

## Resultados

 Las etapas 1, 2 y 3 llevaron a la creación de la aplicación denominada “ *Nasci Bem*”. Con varios cursores especializados en sitios *web* y *mobile*, se utilizó el lenguaje de programación *Javascript* junto con *React Native Framework*. Los colores pastel azul y verde del folleto se mantuvieron en la interfaz. Las ilustraciones del folleto se obtuvieron de *Canva*, utilizando una cuenta PRO, a excepción del logotipo, que fue creado específicamente para este producto. Los íconos de las pestañas se generaron en base a imágenes *Freepik* disponibles en la plataforma *Google*. 

**Figura 1:** - Captura de pantalla de la aplicación “*Nasci Bem*”. Rio das Ostras, RJ, Brasil, 2023

 Al abrir “*Nasci Bem*”, aparece un globo explicativo sobre la tecnología en el centro de la pantalla. Luego de cerrarlo, aparece el logo y una solicitud para elegir entre dos opciones de acceso: “*Profissional de saúde*” (“Profesional de la salud”) o “*Familiar de recém-nascido*” (“Familiar del recién nacido”). Al seleccionar uno de ellos aparecen tres pestañas: “*Cartilha*” (“Folleto”), “Quiz” (“Cuestionario”) y “*Quem somos*” (“Quiénes somos”). El contenido de la pestaña “Folleto” se adapta según el perfil del usuario. 

Ambas pestañas ofrecen contenidos similares, pero con un lenguaje dirigido a cada público: presentación; buenas prácticas; identificación de buena vitalidad; qué se debe hacer inmediatamente después del parto; prácticas que hay que posponer; administración de vitamina K; prevención de la oftalmía neonatal; ejemplos de cuidados sencillos para prevenir muertes evitables; materiales de apoyo; y referencias.

La pestaña “Cuestionario” propone 10 preguntas para que el público evalúe su conocimiento, aclare posibles dudas sobre las prácticas realizadas con los recién nacidos en la maternidad de forma ágil, y les ofrece dos opciones de respuesta (Verdadero o Falso). Después de que se selecciona una opción, la respuesta correcta se resalta en un tono más vibrante, verde para verdadero y rojo para falso. Además, aparece un texto explicativo para cada respuesta, se destaca la respuesta correcta y se la contextualiza.

 Si el usuario elige salir del cuestionario antes de completar todas las preguntas, aparecerá un mensaje: “*Retornar para o menu? Você perderá seu progresso*” (“¿Volver al menú? Perderás tu progreso”). A esto le seguirán dos opciones: “*Ir para o menu*” (“Ir al menú”) y “Cancelar”. Después de que el usuario responda todas las preguntas, se muestra el número de respuestas correctas. Además, la sección “Quiénes somos”, disponible para ambos públicos, destaca a los creadores de la tecnología educativa, el proyecto que dio origen a la aplicación, sus objetivos y *links* de redireccionamiento a otros productos tecnológicos desarrollados por el equipo de investigación. A continuación, se describen las etapas de validación y evaluación de la aplicación (4, 5, 6 y 7). 

En la validación (etapa 4) participaron 22 expertos, con una edad promedio de 42 años, que osciló entre 25 y 70. Todos los participantes eran del sexo femenino y enfermeras, cuatro (18%) tenían título de especialización, 12 (55%) de doctorado y seis (27%) de maestría. En cuanto a las áreas de especialización, seis (27%) eran en Pediatría y Neonatología, cinco (23%) en Pediatría, tres (14%) en Neonatología y 1 (4%) en cada una de las siguientes áreas: Salud del Niño y del Adolescente, Unidad de Terapia Intensiva Neonatal y Pediátrica, Neonatología y Perinatología y Obstetricia. En cuanto al tiempo de experiencia profesional, 20 (91%) trabajaban hace más de cinco años.

 La Tabla 1 presenta la validación de los expertos sobre el contenido y la apariencia, según el IC por ítem y general. 

**Tabla 1 -:** Validación de los expertos sobre el contenido y la apariencia de la primera versión de la aplicación (n = 22). Rio das Ostras, RJ, Brasil, 2023

**Sobre el contenido**			
**Ítem**	**Estoy muy o poco en desacuerdo**	**Estoy muy o poco de acuerdo**	IC* del ítem
1) La aplicación facilita el aprendizaje de los conceptos utilizados y sus aplicaciones.	0	22	1
2) La aplicación es atractiva para las gestantes, puérperas y familiares de recién nacidos y para los profesionales de la salud que trabajan en la maternidad.	1	21	0,95
3) La aplicación brinda ayuda completa.	0	22	1
4) Se aborda información que promueve las prácticas de atención humanizada al recién nacido.	0	22	1
5) Invita y/o fomenta cambios en la población (gestantes, puérperas y familiares de recién nacidos y profesionales de la salud que trabajan en la maternidad).	0	22	1
6) La aplicación es apta para que la utilice cualquier individuo de la población.	4	18	0,82
7) El contenido de la aplicación corresponde al contenido presente en la literatura científica.	0	22	1
8) La aplicación es apropiada para el propósito para el que se creó.	0	22	1
**Sobre la apariencia**			
**Ítem**	**Estoy muy o poco en desacuerdo**	**Estoy muy o poco de acuerdo**	IC* del ítem
1) Las letras tienen un tamaño adecuado.	3	19	0,86
2) La interfaz de la aplicación es atractiva.	1	21	0,95
3) Las imágenes son fáciles de entender.	0	22	1
4) Los colores son adecuados.	0	22	1
5) La cantidad de las preguntas del cuestionario es adecuada.	0	22	1
6) La aplicación parece organizada.	0	22	1
7) Todas las pantallas permiten acceder a los menús y las funciones comunes de la aplicación.	0	22	1
**Índice de Concordancia General = 0,97**			

* IC = Índice de Concordancia

El IC promedio para todos los ítems fue superior a 0,8 (80%), tanto para el contenido como para la apariencia, lo que demuestra que la aplicación recibió una evaluación satisfactoria, y alcanzó un índice promedio general de 0,97 (97%). En los ítems evaluativos hubo una variación de 0,82 (82%) a 1,0 (100%).

En la evaluación (etapa 6) participaron 21 personas del público objetivo, ocho profesionales de la salud de la sala de parto y/o alojamiento conjunto (38%) y 13 familiares de recién nacidos (62%), incluidas gestantes, puérperas y familiares de recién nacidos. Entre los profesionales, dos eran enfermeras (9,5%) y seis técnicos en enfermería (28,6%), con experiencia entre tres meses y 12 años. De los familiares, 11 eran madres (52,4%) y dos tías (9,5%), cinco tenían educación superior (23,8%), siete educación secundaria (33,3%) y uno educación primaria (4,8%). El rango etario osciló entre 19 y 43 años, con una media de 31 años.

 La Tabla 2 , a continuación, presenta la evaluación del público objetivo sobre el contenido, la apariencia y la motivación, con el IC por ítem y general. 

**Tabla 2 -:** Evaluación del público objetivo del contenido, apariencia y motivación de la segunda versión de la aplicación (n = 21). Rio das Ostras, RJ, Brasil, 2023

**Sobre el contenido**			
**Ítem**	**Estoy muy o poco en desacuerdo**	**Estoy muy o poco de acuerdo**	IC* del ítem
1) El lenguaje utilizado en la aplicación es fácil de entender.	0	21	1
2) La información que se da es clara.	0	21	1
3) La aplicación facilita el aprendizaje de las prácticas de atención humanizada en las maternidades.	0	21	1
4)Invita y/o fomenta cambios en la atención que se le brinda al recién nacido en la maternidad.	0	21	1
5) El cuestionario aborda claramente las principales prácticas de atención recomendadas, no recomendadas y recomendadas con criterios.	0	21	1
6) El cuestionario es atractivo.	0	21	1
**Sobre la apariencia**			
**Ítem**	**Estoy muy o poco en desacuerdo**	**Estoy muy o poco de acuerdo**	IC* del ítem
1) Las letras tienen un tamaño adecuado.	0	21	1
2) La interfaz de la aplicación es atractiva.	0	21	1
3) Las imágenes son fáciles de entender.	0	21	1
4) Los colores son adecuados.	0	21	1
5) La aplicación parece organizada.	1	20	0,95
6) Todas las pantallas permiten acceder a los menús y las funciones comunes de la aplicación.	0	21	1
**Sobre la motivación**			
**Ítem**	**Estoy muy o poco en desacuerdo**	**Estoy muy o poco de acuerdo**	IC* del ítem
1) La aplicación brinda ayuda de forma positiva.	0	21	1
2) La aplicación te hizo pensar en prácticas de atención humanizadas.	0	21	1
3) La aplicación te motivó a cambiar tus hábitos sobre la atención que le brindas a los recién nacidos en la maternidad.	0	21	1
**Índice de Concordancia General = 0,99**			

* IC = Índice de Concordancia

El IC promedio para todos los requisitos de contenido, apariencia y motivación superó el 0,8 (80%), lo que indica que la aplicación presentó una evaluación satisfactoria, y alcanzó un valor promedio general de 0,99 (99%). En los ítems evaluativos hubo una variación de 0,95 (95%) a 1,0 (100%).

 La Tabla 3 presenta la validación de los expertos y la evaluación del público objetivo sobre la usabilidad, según el IC por ítem y el Puntaje de Usabilidad general de la escala SUS. 

En la validación de la usabilidad que realizaron los expertos de la primera versión de la aplicación, mediante la escala SUS, el IC para todos los ítems fue igual o mayor a 0,8 (80%) y varió entre 0,82 (82%) y 1,0 (100%), la media fue de 0,92 (92%), lo que indica que la valoración fue satisfactoria. Además, el Puntaje General de Usabilidad según la escala SUS fue 93, varió entre 77,5 y 100, lo que demuestra que tiene el mejor grado de usabilidad posible.

**Tabla 3 -:** Validación de los expertos de la primera versión (n = 22) y evaluación del público objetivo de la segunda versión de la aplicación (n = 21) sobre usabilidad mediante la *System Usability Scale*. Rio das Ostras, RJ, Brasil, 2023

	**Primera versión**			**Segunda versión**		
**Ítem**	**Estoy muy o poco en desacuerdo / No estoy de acuerdo ni en desacuerdo**	**Estoy muy o poco de acuerdo**	IC* del ítem entre los expertos	**Estoy muy o poco en desacuerdo / No estoy de acuerdo ni en desacuerdo**	**Estoy muy o poco de acuerdo**	IC* del ítem entre el público objetivo
1. Usaría esta aplicación con frecuencia.	2	20	0,91	1	20	0,95
2. La aplicación me pareció compleja innecesariamente.	20	2	0,91	17	4	0,8
3. La aplicación me pareció fácil de usar.	0	22	1	0	21	1
4. Creo que necesitaría ayuda del soporte técnico para poder usar esta aplicación.	18	4	0,82	20	1	0,9
5. Me pareció que las diferentes funciones de la aplicación estaban bien integradas.	1	21	0,95	0	21	1
6. Me pareció que había mucha inconsistencia en esta aplicación.	22	0	0,95	20	1	0,9
7. Creo que la mayoría de la gente aprendería a utiliza.	1	21	0,95	1	20	0,95
8. La aplicación me pareció muy lenta.	21	1	0,86	21	0	1
9. Me sentí muy seguro al usar la aplicación.	0	22	1	1	20	0,95
10. Tuve que aprender varias cosas antes de poder seguir usando la aplicación.	20	2	0,86	20	1	0,9
**Índice de Concordancia General entre los expertos = 0,92**						
**Puntaje de Usabilidad entre los expertos = 93**						
**Índice de Concordancia General entre el público objetivo = 0,93**						
**Puntaje de Usabilidad entre el público objetivo = 94**						

* IC = Índice de Concordancia

A pesar de que se obtuvo una evaluación satisfactoria, se incorporaron las sugerencias de los expertos para mejorar la aplicación, considerando su viabilidad. Las sugerencias incluyeron: fuente más grande en los folletos, lenguaje adaptado en el cuestionario para profesionales y familiares, explicación de términos técnicos, ajustes en la redacción, inclusión de referencias, incorporación de datos en “Quiénes somos” y contenido sobre algunas enfermedades y prácticas.

Luego de realizar los ajustes, el público objetivo evaluó la segunda versión de la aplicación, y obtuvo un IC igual o superior a 0,8 (80%) en todos los ítems, varió entre 0,8 (80%) y 1,0 (100%) y el promedio fue de 0,93. (93%), lo que indica que obtuvo nuevamente una evaluación satisfactoria. Además, el Puntaje de Usabilidad general en la escala SUS fue 94, varió entre 60 y 100, y obtuvo nuevamente el mejor grado de usabilidad posible.

 Los ítems sobre contenido, apariencia, motivación y usabilidad de la aplicación “*Nasci Bem*” recibieron excelentes evaluaciones de los expertos y del público objetivo, y se observó que mejoró el IC promedio general de los ítems y el Puntaje de Usabilidad entre la primera y la segunda versión. 

El público objetivo también hizo sugerencias y solicitó que estuviera disponible en las tiendas de aplicaciones, que se incluyera información sobre la administración oral de vitamina K en caso de rechazo de la vía intramuscular y que mejorara la distribución del contenido de los folletos. Después de realizar los ajustes finales, la versión final de la aplicación se lanzará en las tiendas de aplicaciones.

 Por último, los elementos de la escala SUS tienen atributos de usabilidad específicos que tienen significados relevantes y se utilizan para evaluar los aspectos de calidad de un *software* (Tabla 4). 

**Tabla 4:** - Validación de los expertos de la primera versión y evaluación del público objetivo de la segunda versión sobre las características de usabilidad (n = 43). Rio das Ostras, RJ, Brasil, 2023

	**Primera versión**		**Segunda versión**		**Significado**
**Características de la usabilidad**	**Media de los ítems según los expertos**	**Media general**	**Media de los ítems según el público objetivo**	**Media general**	
Sistema fácil de entender	I* 3 (100,0)	96,6	I* 3 (100,0)	94,6	Sistema fácil de usar cuando se utiliza por primera vez
	I* 4 (100,0)		I* 4 (94,0)		
	I* 7 (96,6)		I* 7 (94,0)		
	I* 10 (89,8)		I* 10 (90,5)		
Eficiencia del sistema	I* 5 (97,7)	93,2	I* 5 (96,4)	94,8	Rapidez en la ejecución de las tareas establecidas
	I* 6 (95,5)		I* 6 (90,5)		
	I* 8 (86,4)		I* 8 (97,6)		
Inconsistencias	I* 6 (95,5)	95,5	I* 6 (90,5)	90,5	Ausencia de errores
Facilidad de memorización	I* 2 (92,0)	92,0	I* 2 (86,9)	86,9	Sistema fácil de usar incluso después de un largo período sin usarlo
Satisfacción del usuario	I* 1 (89,8)	95,8	I* 1 (94,0)	94,4	Buen diseño
	I* 4 (100,0)		I* 4 (94,0)		
	I* 9 (97,7)		I* 9 (95,2)		

* I = Ítem

Los resultados revelaron que todos los ítems, desde la primera versión, obtuvieron un puntaje superior a 86 en las características de usabilidad de la aplicación. Esto indica que todos alcanzaron índices clasificados como el mejor posible, lo que la convierte en una tecnología educativa cuyo sistema tiene alta facilidad de comprensión y memorización, además de alta satisfacción y eficiencia, y baja inconsistencia.

## Discusión

El estudio logró satisfactoriamente su objetivo de crear, validar y evaluar una aplicación móvil sobre prácticas de atención humanizada al recién nacido de riesgo habitual en la maternidad. La aplicación está dirigida a profesionales de la salud, gestantes, puérperas y familias de recién nacidos, y obtuvo un índice de concordancia en contenido, apariencia, motivación y usabilidad, tanto por ítem como general, por encima de los valores deseables. Además, la usabilidad, desde la primera versión, alcanzó un nivel clasificado como “el mejor posible”, lo que indica que tiene un excelente potencial de uso para el público objetivo.

 Es importante destacar lo relevante que es crear tecnologías educativas en salud, someterlas a la validación de los expertos y a la evaluación del público objetivo, especialmente las relacionadas con temas de interés para la población y que satisfagan sus necesidades de aprendizaje ^(29)^. A través de este tipo de productos tecnológicos es posible incrementar el acceso a información en salud de calidad y promover la autonomía y el empoderamiento tanto de los profesionales de la salud como de las familias para promover las buenas prácticas de atención a los recién nacidos de riesgo habitual en la maternidad. 

 En función de la evidencia científica se puede ver el desarrollo global que tiene las tecnologías educativas en forma de aplicaciones móviles, con el fin de instruir, informar y orientar. Un estudio creó y evaluó un *Serious Game* centrado en mejorar el cuidado que le brinda la familia al bebé prematuro, y obtuvo resultados que coinciden con los del presente estudio. La evaluación del juego reveló que tenía niveles satisfactorios de contenido, apariencia y usabilidad, y que incentivaba a los participantes durante el proceso de aprendizaje ^(29)^. 

 Otro estudio desarrolló, validó y evaluó una tecnología de extracción de leche materna dirigida a enfermeros que trabajan en agroindustrias. Esa investigación obtuvo un Índice de Validez de Contenido (IVC) de 86,72% en la validación de los expertos y obtuvo un puntaje de evaluación de la usabilidad del juego en forma de aplicación de 83,89 por parte de los enfermeros ^(30)^. Esos índices son inferiores a los obtenidos en el presente estudio, que utilizó la misma escala de evaluación. Otra investigación desarrolló y validó una aplicación móvil para gestantes en control prenatal que aborda preguntas sobre lactancia materna. Este estudio logró un IVC de 0,89 por parte de los expertos, lo que demuestra la confiabilidad que tiene la información y la parte técnica del sistema. Esta aplicación tiene potencial como recurso para promover la salud durante el período embarazo-puerperio ^(13)^.

 Un proyecto creó la aplicación “*Preemie Care*” (“Atención de prematuros”) para orientar a los profesionales de la salud sobre la atención neonatal, y al evaluar su usabilidad con especialistas mediante la escala SUS, alcanzó un puntaje de 88%. Esto indica que tiene una usabilidad general aceptable y excelente ^(18)^, y se acerca al resultado obtenido en el presente estudio, en el que la calificación fue “la mejor posible” en todas las características del sistema. 

 En el proceso de creación, validación y evaluación de la aplicación “ *Descomplicando a amamentação*” (“Simplificando la lactancia”), las etapas de validación y evaluación se realizaron en estudios separados. En la validación de contenido, apariencia y usabilidad mediante el IVC se obtuvo un promedio de 0,96 ^(22)^. Al evaluar la semántica, apariencia y usabilidad mediante el IC y la escala SUS, respectivamente, se alcanzaron valores de 0,99 y 93, lo que indica que tuvo resultados satisfactorios y altamente efectivos para ser utilizado por las familias ^(31)^. Estos resultados son similares a los obtenidos en la presente investigación, que fueron superiores en algunos aspectos. 

 Sólo se encontró una investigación que realizó la validación y evaluación en el mismo estudio ^(30)^. Si bien todos los ítems lograron excelentes resultados, la presente investigación logró un desempeño superior tanto en la validación de los expertos como en la evaluación del público objetivo. Cabe señalar que la mayoría de las aplicaciones móviles que se encontraron dirigidas a esta población se centran en embarazos de alto riesgo y atención específica al recién nacido prematuro. Sin embargo, lo que diferencia este estudio de otros es que el enfoque se centra en los embarazos y recién nacidos de riesgo habitual, por lo que contribuye significativamente a la salud materno infantil. 

 Estudios internacionales han demostrado que el uso de aplicaciones móviles sobre embarazo, parto y nacimiento son eficientes para mejorar el conocimiento de los profesionales de la salud y de los familiares, al brindarle información y empoderar al usuario ^(32) - 33)^. También pueden contribuir a la comunicación entre las familias y los profesionales durante el período prenatal, fortaleciendo la toma de decisiones ^(34)^, lo que destaca la importancia del presente estudio. 

 Para que estas tecnologías sean efectivas, es fundamental considerar los aspectos esenciales que determinan la calidad de un *software*, como se hizo en este estudio al evaluarlo mediate la escala SUS. Esta escala abarca diferentes aspectos del sistema, como la interfaz, el soporte que requiere, la complejidad, entre otros. Por lo tanto, esta herramienta de evaluación tiene alta validez para medir la usabilidad de una aplicación, y se configura como un instrumento de evaluación confiable y robusto ^(27)^. 

 Esta clasificación evaluó la aplicación móvil “*Nasci Bem*” como el dispositivo con la mejor usabilidad posible, y los usuarios consideraron que tiene fácil uso, satisfacción, eficiencia y baja inconsistencia. Por lo tanto, es esencial que las aplicaciones sean sometidas a pruebas de usabilidad antes de ponerlas a disposición del público, dado que es uno de los principales criterios para garantizar que una aplicación móvil sea fácil de usar para los usuarios y alcance los objetivos propuestos ^(35)^. 

Las limitaciones del estudio incluyen que la selección haya sido realizada por conveniencia, lo que restringe el análisis a los participantes seleccionados, y que dependiera del acceso a Internet y a dispositivos móviles, lo que destaca que es necesario que se lleven a cabo estudios adicionales con diferentes públicos y otros enfoques metodológicos.

 La disponibilidad de la aplicación “*Nasci Bem*” puede reducir prácticas inapropiadas, como la separación de la madre y el bebé al nacer, el pinzamiento temprano del cordón umbilical y la aspiración de las vías respiratorias y del tracto gástrico en la sala de partos, así como también fomentar la adopción de medidas apropiadas como la lactancia materna y el contacto temprano piel con piel. La tecnología reúne prácticas de atención humanizada al recién nacido, al agrupar recomendaciones actualizadas en un solo lugar, empoderar al público objetivo, que puede acceder a esta información en cualquier momento, y promover una mayor autonomía. El objetivo es reducir la morbimortalidad neonatal e infantil y ampliar la salud digital. 

## Conclusión

 Los profesionales de la salud y los familiares que participaron de las etapas metodológicas de este estudio consideraron que la aplicación “*Nasci Bem*” era comprensible, relevante, pertinente, fácil de usar, de baja inconsistencia y que tenía un excelente potencial de uso como tecnología educativa en salud. 

Esta innovadora herramienta surge como una herramienta facilitadora para los profesionales de la salud, que brinda capacitación en prácticas humanizadas para los recién nacidos. Además, les ofrece apoyo a las gestantes, puérperas y sus familias, al posibilitar la construcción del conocimiento desde la atención prenatal sobre las mejores prácticas de cuidado del bebé, y hacer el aprendizaje práctico y accesible.

 La aplicación está disponible para descarga gratuita en el sistema operativo *Android*, en la plataforma *Google Play Store*, y próximamente también estará disponible para iOS, en *Apple Store*. 

# Translation: Aplicativo “Nasci Bem” para profissionais de saúde e familiares de recém-nascidos: construção, validação e avaliação*

**DOI:** 10.1590/1518-8345.7264.4261

**Published:** 2024-11-15

**Authors:** Ingrid Lucchese, Fernanda Garcia Bezerra Góes, Maithê de Carvalho e Lemos Goulart, Maria da Anunciação Silva, Aline Cerqueira Santos Santana da Silva, Liliane Faria da Silva

**Affiliations:** ^1^ Universidade Federal Fluminense, Instituto de Humanidades e Saúde, Rio das Ostras, RJ, Brasil.; ^2^ Bolsista do Conselho Nacional de Desenvolvimento Científico e Tecnológico (CNPq), Brasil.; ^3^ Universidade Federal do Estado do Rio de Janeiro, Rio de Janeiro, RJ, Brasil.; ^4^ Universidade Federal Fluminense, Escola de Enfermagem Aurora de Afonso Costa, Niterói, RJ, Brasil.

**Keywords:** Aplicativos Móveis, Alojamento Conjunto, Maternidades, Recém-nascido, Salas de Parto, Tecnologia Educacional

## Abstract

construir, validar e avaliar um aplicativo para dispositivos móveis sobre práticas assistenciais humanizadas ao recém-nascido de risco habitual na maternidade, destinado a profissionais de saúde e familiares.

estudo metodológico em sete etapas: revisão de literatura; organização do conteúdo; construção do aplicativo; validação por experts; adequação após validação; avaliação pelo público-alvo; e adequação após avaliação. Participaram 22 experts da validação e 21 pessoas do público-alvo na avaliação. Os instrumentos abordaram conteúdo, aparência e/ou motivação, com a *System Usability Scale* para usabilidade. Calcularam-se: Índice de Concordância e Escore de Usabilidade.

na validação, o aplicativo alcançou Índice de Concordância de 0,97; variando de 0,82 a 1,0 entre os itens analisados. Na avaliação, o índice foi de 0,99; variando de 0,95 a 1,0. Na usabilidade da primeira versão, o Índice de Concordância foi de 0,92 e o Escore de Usabilidade de 93. Já na segunda, o índice foi de 0,93 e escore de 94, indicando usabilidade melhor alcançável desde a primeira versão.

o aplicativo “Nasci Bem” demonstrou ser compreensível, relevante, pertinente, eficiente, de fácil utilização e baixa inconsistência, e com excelente potencial de uso como tecnologia educacional para profissionais e familiares.

## Destaques

**(1)** O estudo construiu, validou e avaliou o aplicativo móvel “Nasci Bem”.

**(2)** Participaram da pesquisa 22 experts e 21 profissionais de saúde e familiares.

**(3)** A validação e a avaliação apresentaram índices altos de concordância e usabilidade.

**(4)** A tecnologia educacional é compreensível, relevante, eficiente e de fácil utilização.

**(5)** O aplicativo “Nasci Bem” colabora para a saúde digital.

## Introdução

 O Programa de Humanização do Pré-natal e Nascimento brasileiro visa aprimorar o acompanhamento pré-natal e a assistência ao parto e puerpério para mãe-bebê, promovendo uma abordagem humanizada ao parto como uma experiência verdadeiramente humana ^(1)^. Conforme as diretrizes globais de assistência a gestantes saudáveis da Organização Mundial de Saúde (OMS), a intervenção imediata da equipe de saúde em situações de risco habitual não é recomendada, enfatizando a singularidade de cada mulher e seu processo de parto para uma experiência positiva. É fundamental envolvê-las nas decisões sobre os cuidados recebidos, mesmo quando intervenções profissionais forem necessárias ^(2)^. 

 No Brasil, o modelo predominante de atendimento durante o parto ainda é tecnocrático, focado nos profissionais de saúde e propenso a intervenções inadequadas ^(3)^. Portanto, práticas humanizadas recomendadas pela OMS, como contato pele a pele precoce, que estimula o aleitamento materno, previne a hipotermia neonatal e fortalece o vínculo entre mãe e bebê; amamentação na primeira hora de vida, que reduz o desmame precoce e promove a saúde cardiorrespiratória; clampeamento oportuno do cordão umbilical, que diminui a anemia nos primeiros meses de vida; e presença do acompanhante na sala de parto, reconhecida como promotora dessas boas práticas, não estão sendo adotadas efetivamente ^(1 - 3)^. 

 É necessário sensibilizar os profissionais de saúde sobre as práticas humanizadas na maternidade, visando proporcionar conforto e segurança e reduzir cesarianas desnecessárias ^(4)^, as quais dificultam, mas não impedem, o contato pele a pele e a amamentação precoces, devido à anestesia e à posição do leito em grau zero ^(5)^. Ademais, mulheres submetidas à cesariana liberam menos ocitocina, hormônio crucial para a apojadura, imediatamente após o parto ^(6)^, e sentem mais dor e desconforto pós-cirúrgico ^(7)^. Porém, a taxa de cesáreas no Brasil excede amplamente os índices sugeridos pela OMS de 10% a 15% ^(8)^, situando-se em torno de 55% ^(9)^, limitando a aplicação efetiva das práticas humanizadas. Todavia, as práticas ideais para a assistência ao bebê após uma cesárea devem ser as mesmas do parto vaginal ^(4)^.

 Vários fatores favorecem a adesão às práticas humanizadas ao recém-nascido de risco habitual na maternidade, especialmente o parto vaginal, a boa vitalidade e o contato pele a pele precoce, que facilita o clampeamento oportuno do cordão umbilical e a amamentação na primeira hora de vida ^(1 - 3)^. Embora alguns recém-nascidos requeiram intervenções, em muitos centros hospitalares, práticas como a separação do binômio, o uso de oxigênio inalatório, a aspiração oronasofaríngea, gástrica e traqueal ^(1, 10)^ e o clampeamento precoce ou imediato do cordão umbilical ^(4)^ ainda persistem, mesmo em recém-nascidos saudáveis ^(3)^. Às vezes, essas intervenções são preferidas, mesmo sem condições patológicas justificáveis ^(10)^.

 Os profissionais de saúde demonstram conhecimento sobre as práticas humanizadas, o que é considerado um ponto de partida fundamental para sua implementação, conforme a literatura ^(11)^. Mas sua efetiva adesão requer sensibilização de gestores e profissionais, apoio institucional e atualização baseada em evidências. Ademais, recomenda-se compartilhar orientações sobre cuidados pós-natais seguros desde o pré-natal até a transição da maternidade para o domicílio ^(12)^, visando capacitar gestantes, puérperas e familiares.

 As tecnologias educacionais em saúde, integradas ao cotidiano de pacientes e profissionais, têm o potencial de promover a saúde mediante informações sobre prevenção e tratamento de agravos à saúde ^(13)^. Conforme a 33ª Pesquisa Anual do Uso de Tecnologias de Informação da Fundação Getúlio Vargas, em 2022, o Brasil possuía 447 milhões de dispositivos digitais (computadores, *notebooks*, *tablets* e *smartphones*), mais de dois por habitante ^(14)^. Dada a ampla presença desses dispositivos, notadamente os *smartphones*, o uso de tecnologias educacionais é um recurso promissor para o enfermeiro atuar dinamicamente como educador em saúde. Globalmente, a saúde digital é reconhecida como uma ferramenta eficaz na promoção de informações de saúde, alcançando diversos públicos ^(15 - 16)^. 

 Em 2020, o Brasil, alinhado às recomendações globais, lançou o Plano de Ação de Saúde Digital 2020 – 2028, delineando estratégias para alcançar a Visão de Saúde Digital. Este plano propôs a criação de uma plataforma integrada para promover a troca de dados na Rede de Atenção à Saúde, com a expectativa de sua implementação em todas as 27 unidades federativas até o final de 2023 ^(17)^ Esse desenvolvimento respalda a concepção de ferramentas de saúde digital direcionadas para a educação em saúde, facilitando o cuidado contínuo, independentemente do momento ou local.

 Na literatura, identificaram-se pesquisas que criaram aplicativos na área, um para gestantes em pré-natal, visando promover saúde durante o período gravídico-puerperal ^(13)^ e outro voltado para profissionais de saúde para avaliar riscos à saúde neonatal e difundir práticas de cuidado para recém-nascidos ^(18)^. Contudo, lacunas de conhecimentos foram evidentes pela escassez de estudos internacionais e nacionais sobre aplicativos abordando práticas assistenciais humanizadas ao recém-nascido de risco habitual na maternidade, voltados para profissionais e familiares simultaneamente. Surgiu, assim, a intenção de produzir um aplicativo móvel nessa vertente, como tecnologia educacional para apoiar decisões assertivas dos profissionais de saúde e proporcionar conhecimento prático e acessível aos familiares desde o pré-natal. 

A presente pesquisa visa colaborar para o avanço do conhecimento científico e da saúde digital, apresentando integralmente o processo da concepção à avaliação de um aplicativo inovador que reúne conteúdos atualizados sobre as práticas assistenciais ao recém-nascido: recomendadas, recomendadas com critério e não recomendadas, de forma móvel, acessível e de acesso livre, o reforça a relevância deste estudo.

Assim, o objetivo do estudo foi construir, validar e avaliar um aplicativo para dispositivos móveis sobre práticas assistenciais humanizadas ao recém-nascido de risco habitual na maternidade destinado a profissionais de saúde e familiares.

## Método

### Tipo do estudo

 Estudo metodológico de abordagem quantitativa que construiu, validou e avaliou uma tecnologia educacional em formato de aplicativo móvel em sete etapas: 1ª) revisão de literatura; 2ª) organização do conteúdo; 3ª) construção do aplicativo (*design* e desenvolvimento); 4ª) validação do aplicativo por experts; 5ª) adequação após validação; 6ª) avaliação do aplicativo pelo público-alvo; 7ª) adequação após avaliação. Os estudos metodológicos são geralmente não experimentais e focam na criação de novos materiais educativos ^(19 - 20)^ . 

### Cenário

O estudo ocorreu em ambiente virtual.

### Período

O desenvolvimento do aplicativo começou em janeiro de 2023 e a coleta de dados para validação e avaliação ocorreu entre agosto e novembro de 2023.

### População

 A população do estudo incluiu *experts* (profissionais de saúde com experiência em neonatologia, pediatria ou obstetrícia) e público-alvo (profissionais de saúde de maternidades e gestantes, puérperas e familiares de recém-nascidos). 

### Critérios de seleção

 Para seleção dos *experts* utilizaram-se os critérios de inclusão: profissionais de saúde com experiência em neonatologia, pediatria ou obstetrícia; e aplicaram-se os critérios de Fehring ^(20)^, que atribui uma pontuação de um a cinco pontos, considerando: participação em eventos científicos nos últimos dois anos sobre a temática (um ponto), experiência mínima de cinco anos na área (dois pontos), publicação em periódicos indexados sobre o tema (dois pontos), título de especialista (três pontos), título de mestre (quatro pontos) e título de doutor (cinco pontos). Para conferência do escore, foram verificados os dados nos currículos da Plataforma Lattes de cada profissional selecionado, excluindo aqueles que não atingissem a pontuação mínima de cinco pontos. Além disso, como critério de exclusão aplicou-se a profissionais com atividades exclusivamente administrativas. 

 O público-alvo foi selecionado mediante os critérios de inclusão: profissionais de saúde atuantes na sala de parto e/ou no Alojamento Conjunto, gestantes, puérperas e familiares de recém-nascidos maiores de 18 anos, com acesso à internet. Os critérios de exclusão envolveram profissionais de saúde que desempenhassem funções como auxiliares de enfermagem, não realizassem assistência direta ao binômio mãe-bebê ou se dedicassem apenas a atividades administrativas. Além disso, gestantes, puérperas ou familiares de recém-nascidos com limitações físicas e/ou mentais para responder a formulários *online* ou que fossem analfabetos. 

### Definição da amostra

 Os participantes foram convidados mediante seleção por conveniência e não probabilística. A amostra consistiu em 22 experts e 21 indivíduos do público-alvo, que devolveram suas avaliações no prazo estipulado de dez dias. Essa distribuição segue a recomendação da literatura sobre a elaboração de tecnologias educacionais, que indica no mínimo nove participantes para cada grupo de avaliadores ^(21)^. No público-alvo, oito eram profissionais de saúde e 13 eram gestantes, puérperas e familiares de recém-nascidos. 

### Variáveis do estudo

A primeira seção do instrumento de validação pelos experts incluiu perguntas fechadas para caracterização dos participantes, como idade, sexo, formação e área de atuação profissional, qualificações e tempo de experiência. No instrumento de avaliação do público-alvo, foram incluídos aspectos como idade, sexo, nível de escolaridade, profissão, tipo de envolvimento (profissional de saúde ou familiar de recém-nascido), tempo de atuação na maternidade (para profissionais de saúde) e relação com o recém-nascido (para familiares). A segunda seção abordou questões específicas relacionadas à análise da tecnologia educacional.

### Instrumentos utilizados para a coleta das informações

 O processo de coleta de dados para validação e avaliação do aplicativo móvel foi conduzido por meio do *Google Forms* na plataforma *Google*. A página da pesquisa *online* continha informações detalhadas sobre o projeto, o Registro de Consentimento Livre e Esclarecido (RCLE) disponível para *download* pelos participantes e o formulário eletrônico para a coleta de dados. 

 O instrumento de validação do aplicativo pelos *experts*, previamente validado, incluiu oito questões sobre o conteúdo e sete sobre a aparência. Para a avaliação pelo público-alvo, utilizaram-se seis questões sobre o conteúdo, seis sobre a aparência e três sobre a motivação, mediante um instrumento também validado anteriormente. Os itens foram pontuados em uma escala Likert de um a quatro pontos, representando respectivamente: “discordo muito” (um ponto), “discordo pouco” (dois pontos), “concordo pouco” (três pontos) e “concordo muito” (quatro pontos) ^(22)^. 

 Sequencialmente, ambos os grupos de avaliadores utilizaram a *System Usability Scale* (SUS) ^(23)^, composta por dez itens. Essa escala utiliza uma pontuação de um a cinco pontos, representando, respectivamente: “discordo muito” (um ponto), “discordo pouco” (dois pontos), “nem concordo, nem discordo” (três pontos), “concordo pouco” (quatro pontos) e “concordo muito” (cinco pontos) ^(24)^. Além disso, ao final dos instrumentos, foi fornecido um espaço para sugestões e comentários para aprimorar a tecnologia educacional em saúde. 

### Coleta de dados

A primeira etapa, a revisão de literatura do tipo narrativa, teve como propósito identificar, reunir e sintetizar informações sobre práticas assistenciais recomendadas, recomendadas com critério e não recomendadas para o cuidado ao recém-nascido de risco habitual na maternidade. Embasada em autores especialistas na área, documentos do Fundo das Nações Unidas para a Infância (UNICEF), da OMS, do Ministério da Saúde (MS) do Brasil e da Sociedade Brasileira de Pediatria (SBP), a revisão buscou publicações dos últimos dez anos, incluindo artigos, manuais, guias, diretrizes e resoluções relacionadas à temática do estudo. Essa busca foi realizada por meio da consulta direta aos sites dessas organizações, reforçando a confiabilidade e atualidade das informações coletadas, envolvendo termos como “Recém-nascido”, “Maternidade”, “Salas de Parto” e “Alojamento Conjunto”.

 Na segunda etapa, correspondente à organização do conteúdo, partiu-se das evidências científicas encontradas na literatura para criar uma matriz de análise com os principais achados nos sites das organizações científicas citadas. A partir da identificação dessas práticas, foram extraídas as condutas essenciais a serem aplicadas na sala de parto e no Alojamento Conjunto, com o intuito de promover práticas assistenciais humanizadas. Com base nessa organização, a equipe desenvolveu os conteúdos para a tecnologia, incluindo textos, imagens e *links*. O objetivo era criar um roteiro para orientar a construção do aplicativo de maneira criativa, atrativa, intuitiva, detalhada, motivadora e eficaz. 

 Na terceira etapa, referente à criação do aplicativo móvel, foi realizada a primeira reunião entre a equipe do estudo e um desenvolvedor especializado em Ciência da Computação, que possui uma empresa de serviços na área. Destaca-se que o desenvolvimento do aplicativo, passo determinante de efetivação, deve ser realizado por profissionais especialistas, com a supervisão das pesquisadoras, como indica outro estudo ^(25)^. Durante essa reunião inicial, foram discutidas ideias para o design instrucional e a estrutura de navegação, adaptada na forma de um aplicativo de *download* gratuito. A interface foi planejada para garantir interatividade, autonomia e acessibilidade em todos os comandos. 

 Em um intervalo de 23 dias, ocorreu uma segunda reunião, na qual o desenvolvedor apresentou uma versão preliminar do aplicativo em formato *Android Application Pack* (APK). Essa versão já continha as abas com o material educativo, além das cores e fontes pré-definidas, permitindo ajustes conforme preferência da equipe. Posteriormente, foi realizada uma nova etapa de inserção de dados na ferramenta educacional, resultando na criação das interfaces: menu principal, cartilhas, quiz e informações sobre cada perfil de usuário, o que originou a primeira versão do aplicativo. 

 Nas etapas quatro e seis, a coleta de dados foi realizada por meio de convites via *WhatsApp*, *e-mails* e *Instagram*, convidando os participantes a validar ou avaliar o aplicativo móvel. Para preservar a privacidade, não foram utilizadas listas identificáveis de contatos. O convite incluía uma breve explicação do projeto, identificação das pesquisadoras, um *link* para a pesquisa e o aplicativo em formato APK para *download* em *smartphones Android*. Foi estabelecido um prazo de 10 dias para a resposta, com uma média de trinta minutos para analisar o aplicativo e preencher o formulário. 

Devido à amostra ser não probabilística, tipo consecutiva, formada ao longo do prazo estipulado pelos pesquisadores, a coleta de dados transcorreu sem dificuldades, com a maioria dos experts e indivíduos do público-alvo convidados respondendo prontamente ao material, sem recusas ou atrasos. Ressalta-se que, devido à natureza da comunicação via mídias sociais, alguns convites enviados não foram respondidos, por isso, foram distribuídos mais convites que o limite mínimo estabelecido, sendo aproximadamente 25 para experts e 30 para o público-alvo.

 Destaca-se que a validação da primeira versão do aplicativo foi conduzida por *experts* na etapa quatro. Somente após as adaptações sugeridas e efetuadas na etapa cinco, avançou-se para a avaliação da segunda versão junto ao público-alvo na etapa seis. Assim, mesmo após essas fases, pequenas modificações ainda se mostraram necessárias para a elaboração da versão final do aplicativo, concluída na sétima e última etapa. 

### Tratamento e análise dos dados

 O estudo analisou os dados quantitativamente, usando o Índice de Concordância (IC) para avaliar os resultados dos *experts* e do público-alvo, considerando as respostas classificadas como três e quatro (concordo pouco e concordo muito), divididas pelo total de respostas. Por esse índice, os itens sobre conteúdo, aparência, motivação e usabilidade, que atingiram uma porcentagem de concordância entre os participantes, igual ou superior a 80%, conforme recomendações da literatura ^(26)^, foram considerados válidos. 

 O cálculo do Escore de Usabilidade da escala SUS foi gerado pela soma das pontuações individuais de cada item. Para itens ímpares, um ponto é subtraído ao valor dado àquela resposta. Para itens pares, o cálculo é feito subtraindo o valor da resposta do total de cinco pontos. Os valores obtidos para itens pares e ímpares são somados e multiplicados por 2,5 para obter o escore total de usabilidade, que varia entre zero e 100 pontos ^(23)^. Vale ressaltar que uma pontuação no Escore de Usabilidade geral da escala SUS entre zero e 25 indica um grau de usabilidade pior alcançável; de 26 a 39 é considerado ruim; 40 a 52 é aceitável; 53 a 74 é bom; 75 a 85 é excelente e 86 a 100 é o melhor alcançável ^(27)^.

 Pela escala SUS, é possível avaliar ainda cinco aspectos-chave para a usabilidade de um aplicativo: 1) facilidade de conhecimento do sistema – itens 3, 4, 7 e 10; 2) eficiência do sistema – itens 5, 6 e 8; 3) inconsistências – item 6; 4) facilidade de memorização – item 2; 5) satisfação do usuário – itens 1, 4 e 9. A pontuação das respostas de cada participante dos itens de cada domínio foi multiplicada por 25 para pontuar as características de usabilidade numa escala de zero a 100. Em seguida, tanto na fase de validação quanto na avaliação, foi calculada a média geral dos escores para cada questão, somando-se os pontos atribuídos por todos os participantes e dividindo pelo número total de respondentes. Posteriormente, foi calculada a média dos itens relacionados às características específicas de cada domínio. Para tal, somaram-se os pontos atribuídos a todos os itens do domínio e dividiu-se pelo total de itens correspondentes ^(27 - 28)^. Itens que não atingissem a marca aceitável seriam revistos. 

### Aspectos éticos

Para atender às diretrizes éticas estabelecidas pela Resolução nº 466/2012 do Conselho Nacional de Saúde, este estudo foi aprovado pelo Comitê de Ética em Pesquisa (CEP) da Universidade Federal Fluminense (UFF) com parecer número 5.900.588 e CAAE: 66077222.8.0000.5243. Além disso, foi fornecido aos participantes o RCLE.

## Resultados

 As etapas 1, 2 e 3 resultaram na construção do aplicativo denominado “Nasci Bem”. Com diversos cursores de especialização em *web* e *mobile*, a linguagem de programação utilizada foi *Javascript* em conjunto com *Framework React Native*. As cores em tons pastéis de azul e verde da cartilha foram mantidas na interface. As ilustrações para a cartilha foram obtidas do Canva, mediante uma conta PRO, com exceção do logo, criado especificamente para este produto. Os ícones das abas foram gerados com base em imagens *Freepik* disponíveis na plataforma *Google*. 

Ao abrir o “Nasci Bem”, surge um balão explicativo sobre a tecnologia no centro da tela. Após fechá-lo, o logo é exibido com uma solicitação para escolher entre duas opções de acesso: “Profissional de saúde” ou “Familiar de recém-nascido”. Ao selecionar uma delas, surgem três abas: “Cartilha”, “Quiz” e “Quem somos”. O conteúdo da aba “Cartilha” é adaptado conforme o perfil do usuário.

Ambas as abas oferecem conteúdos similares, porém com a linguagem voltada para cada público: apresentação; boas práticas; identificação de boa vitalidade; o que deve ser feito imediatamente após o parto; práticas a serem adiadas; administração de vitamina K; prevenção da oftalmia neonatal; exemplos de cuidados simples para prevenir mortes evitáveis; materiais de apoio; referências.

**Figura 1 -:** Captura de telas do aplicativo “Nasci Bem”. Rio das Ostras, RJ, Brasil, 2023

A aba “Quiz” propõe dez questões para o público avaliar seu conhecimento, elucidando possíveis dúvidas sobre as práticas realizadas com os recém-nascidos na maternidade de forma ágil, oferecendo duas opções de resposta (verdadeiro ou falso). Após selecionar uma opção, a resposta correta é destacada com uma tonalidade mais vibrante, verde para verdadeira e vermelha para falsa. Além disso, é exibido um texto explicativo para cada resposta, evidenciando a correta e contextualizando-a.

 Se o usuário optar por sair do *quiz* antes de finalizar todas as questões, uma mensagem surgirá: “Retornar para o menu? Você perderá seu progresso.” Isso será seguido pelas opções “Ir para o menu” e “Cancelar”. Após responder a todas as perguntas, a quantidade de respostas corretas será exibida. Além disso, a seção “Quem somos”, disponível para ambos os públicos, destaca as idealizadoras da tecnologia educacional, o projeto que originou o aplicativo, seus objetivos e *links* para redirecionamento a outros produtos tecnológicos desenvolvidos pela equipe de pesquisa. As etapas de validação e avaliação do aplicativo (4, 5, 6 e 7) são descritas a seguir. 

Na validação (etapa 4), 22 experts participaram, com uma média de idade de 42 anos, variando entre 25 e 70 anos. As participantes eram todas do sexo feminino e enfermeiras, com quatro (18%) possuindo título de especialização, 12 (55%) de doutorado e seis (27%) de mestrado. Quanto às áreas das especializações, seis (27%) eram em Pediatria e Neonatologia, cinco (23%) em Pediatria, três (14%) em Neonatologia, e uma (4%) em cada uma das seguintes áreas: Saúde da Criança e do Adolescente, Unidade de Terapia Intensiva Neonatal e Pediátrica, Neonatologia e Perinatologia, e Obstetrícia. Em relação ao tempo de experiência profissional, 20 (91%) atuavam há mais de cinco anos.

 A Tabela 1 apresenta a validação das *experts* quanto ao conteúdo e à aparência, segundo o IC por item e global. 

**Tabela 1 -:** Validação das *experts* quanto ao conteúdo e à aparência da primeira versão do aplicativo (n = 22). Rio das Ostras, RJ, Brasil, 2023

**Quanto ao conteúdo**			
**Item**	**Discordo muito ou pouco**	**Concordo muito ou pouco**	IC* do item
1) O aplicativo facilita a aprendizagem dos conceitos usados e suas aplicações.	0	22	1
2) O aplicativo é atrativo para gestantes, puérperas e familiares de recém-nascidos e profissionais de saúde que atuam na maternidade.	1	21	0,95
3) O aplicativo fornece ajuda de forma completa.	0	22	1
4) São abordadas informações que promovem práticas assistenciais humanizadas ao recém-nascido.	0	22	1
5) Convida e/ou instiga às mudanças da população (gestantes, puérperas e familiares de recém-nascidos e profissionais de saúde que atuam na maternidade)	0	22	1
6) O aplicativo é adequado ao uso por qualquer indivíduo da população.	4	18	0,82
7) O conteúdo do aplicativo corresponde ao conteúdo presente em literaturas científicas.	0	22	1
8) O aplicativo é apropriado para a proposta a que se destina.	0	22	1
**Quanto à aparência**			
**Item**	**Discordo muito ou pouco**	**Concordo muito ou pouco**	IC* do item
1) As letras estão em um tamanho adequado.	3	19	0,86
2) A interface do aplicativo é atraente.	1	21	0,95
3) As imagens são de fácil compreensão.	0	22	1
4) As cores estão adequadas.	0	22	1
5) As perguntas do quis estão em quantidade adequada.	0	22	1
6) O aplicativo parece organizado.	0	22	1
7) Todas as telas mantêm acessíveis menus e funções comuns do aplicativo.	0	22	1
**Índice de Concordância Global = 0,97**			

* IC = Índice de Concordância

O IC médio de todos os quesitos foi superior a 0,8 (80%), tanto para o conteúdo quanto para a aparência, demonstrando que o aplicativo recebeu avaliação satisfatória, alcançando índice global médio de 0,97 (97%). Nos itens avaliativos, houve variação de 0,82 (82%) a 1,0 (100%).

Na avaliação (etapa 6), participaram 21 pessoas do público-alvo, sendo oito profissionais de saúde da sala de parto e/ou alojamento conjunto (38%) e 13 familiares de recém-nascidos (62%), dentre gestantes, puérperas e familiares de recém-nascidos. Entre os profissionais, duas eram enfermeiras (9,5%) e seis técnicos de enfermagem (28,6%), com experiência variando de três meses a 12 anos. Dos familiares, 11 eram mães (52,4%) e duas eram tias (9,5%), sendo cinco com ensino superior (23,8%), sete com ensino médio (33,3%) e uma com ensino fundamental (4,8%). A faixa etária variou de 19 a 43 anos, com média de 31 anos.

 A Tabela 2 abaixo apresenta a avaliação do público-alvo quanto ao conteúdo, à aparência e à motivação, mediante o IC por item e global. 

**Tabela 2 -:** Avaliação do público-alvo quanto ao conteúdo, à aparência e à motivação da segunda versão do aplicativo (n = 21). Rio das Ostras, RJ, Brasil, 2023

**Quanto ao conteúdo**			
**Item**	**Discordo muito ou pouco**	**Concordo muito ou pouco**	IC* do item
1) A linguagem utilizada no aplicativo é fácil de entender.	0	21	1
2) As informações são dadas com clareza.	0	21	1
3) O aplicativo facilita no aprendizado das práticas assistenciais humanizadas na maternidade.	0	21	1
4) Convida e/ou atrai às mudanças no cuidado prestado ao recém-nascido na maternidade.	0	21	1
5) O *quiz* aborda com clareza as principais práticas assistenciais recomendadas, não recomendadas e recomendadas com critérios.	0	21	1
6) O *quiz* é atrativo.	0	21	1
**Quanto à aparência**			
**Item**	**Discordo muito ou pouco**	**Concordo muito ou pouco**	IC* do item
1) As letras estão em um tamanho adequado.	0	21	1
2) A interface do aplicativo é atraente.	0	21	1
3) As imagens são de fácil compreensão.	0	21	1
4) As cores estão adequadas.	0	21	1
5) O aplicativo parece organizado.	1	20	0,95
6) Todas as telas mantêm acessíveis menus e funções comuns do aplicativo.	0	21	1
**Quanto à motivação**			
**Item**	**Discordo muito ou pouco**	**Concordo muito ou pouco**	IC* do item
1) O aplicativo fornece ajuda de forma positiva	0	21	1
2) O aplicativo levou você a pensar sobre práticas assistenciais humanizadas.	0	21	1
3) O aplicativo te motivou a mudar seus hábitos a respeito do cuidado prestado ao recém-nascido na maternidade.	0	21	1
**Índice de Concordância Global = 0,99**			

* IC = Índice de Concordância

O IC médio para todos os quesitos de conteúdo, aparência e motivação ultrapassou 0,8 (80%), indicando que o aplicativo apresentou avaliação satisfatória, alcançando valor médio global de 0,99 (99%). Nos itens avaliativos, houve variação de 0,95 (95%) a 1,0 (100%).

 A Tabela 3 apresenta a validação das *experts* e a avaliação do público-alvo quanto à usabilidade, conforme o IC por item e o Escore de Usabilidade geral da escala SUS. 

Na validação da usabilidade através da escala SUS pelas experts na primeira versão do aplicativo, o IC para todos os itens foi igual ou superior a 0,8 (80%), variando de 0,82 (82%) a 1,0 (100%), com uma média de 0,92 (92%), indicando uma avaliação satisfatória. Além disso, o Escore de Usabilidade geral pela escala SUS foi de 93, variando entre 77,5 e 100, demonstrando um grau melhor alcançável de usabilidade.

**Tabela 3 -:** Validação das *experts* da primeira versão (n = 22) e avaliação do público-alvo da segunda versão do aplicativo (n = 21) quanto à usabilidade por meio da *System Usability Scale.* Rio das Ostras, RJ, Brasil, 2023

	**Primeira versão**			**Segunda versão**		
**Item**	**Discordo muito ou pouco/ Não concordo nem discordo**	**Concordo muito ou pouco**	IC* do item experts	**Discordo muito ou pouco/ Não concordo nem discordo**	**Concordo muito ou pouco**	IC* do item público-alvo
1. Eu usaria esse aplicativo com frequência	2	20	0,91	1	20	0,95
2. Eu achei o aplicativo. desnecessariamente complexo.	20	2	0,91	17	4	0,8
3. Eu achei o aplicativo fácil para usar.	0	22	1	0	21	1
4. Eu acho que precisaria do apoio de um suporte técnico para ser possível usar este aplicativo.	18	4	0,82	20	1	0,9
5. Eu achei que as diversas funções do aplicativo foram bem integradas.	1	21	0,95	0	21	1
6. Eu achei que houve muita inconsistência neste aplicativo.	22	0	0,95	20	1	0,9
7. Eu imaginaria que a maioria das pessoas aprenderia a usar.	1	21	0,95	1	20	0,95
8. Eu achei o aplicativo muito pesado para uso.	21	1	0,86	21	0	1
9. Eu me senti muito confiante usando o aplicativo.	0	22	1	1	20	0,95
10. Eu precisei aprender uma série de coisas antes que eu pudesse continuar a utilizar o aplicativo.	20	2	0,86	20	1	0,9
**Índice de Concordância Global** experts **= 0,92**						
**Escore de Usabilidade** experts **= 93**						
**Índice de Concordância Global público-alvo = 0,93**						
**Escore de Usabilidade público-alvo = 94**						

* IC = Índice de Concordância

 Apesar da avaliação satisfatória, as sugestões das *experts* foram incorporadas ao aprimoramento do aplicativo, considerando a viabilidade. As sugestões atendidas incluíram: fonte maior em cartilhas, linguagem adaptada no quiz para profissionais e familiares, explicação de termos técnicos, ajustes na redação, inclusão de referências, acréscimo de dados em “Quem somos” e conteúdo sobre algumas doenças e práticas. 

Após os ajustes, o público-alvo avaliou a segunda versão do aplicativo, obtendo-se IC de todos os itens igual ou superior a 0,8 (80%), variando entre 0,8 (80%) a 1,0 (100%), com uma média de 0,93 (93%), indicando novamente uma avaliação satisfatória. Ademais, o Escore de Usabilidade geral pela escala SUS foi de 94, variando entre 60 e 100, classificando-se novamente com um grau melhor alcançável de usabilidade.

 Os itens de conteúdo, aparência, motivação e usabilidade do aplicativo “Nasci Bem” receberam excelentes avaliações das *experts* e do público-alvo, mostrando melhorias no IC médio global dos itens e no Escore de Usabilidade entre a primeira e a segunda versões. 

Como sugestões do público-alvo, solicitou-se a disponibilidade nas lojas de aplicativos, a inclusão de informações sobre administração oral de vitamina K em caso de recusa da via intramuscular e o aprimoramento na distribuição do conteúdo das cartilhas. Após os ajustes finais, a versão final do aplicativo será lançada nas lojas de aplicativos.

 Por último, os elementos da escala SUS possuem atributos específicos de usabilidade que têm significados relevantes, utilizados para avaliar os aspectos de qualidade de um *software* (Tabela 4). 

**Tabela 4 -:** Validação das *experts* da primeira versão e avaliação do público-alvo da segunda versão quanto às características de usabilidade (n = 43). Rio das Ostras, RJ, Brasil, 2023

	**Primeira versão**		**Segunda versão**		**Significado**
**Características da usabilidade**	**Média dos itens entre** experts	**Média geral**	**Média dos itens entre público-alvo**	**Média geral**	
Facilidade de conhecimento do sistema	I* 3 (100,0)	96,6	I* 3 (100,0)	94,6	Sistema de fácil utilização quando utilizado pela primeira vez
	I* 4 (100,0)		I* 4 (94,0)		
	I* 7 (96,6)		I* 7 (94,0)		
	I* 10 (89,8)		I* 10 (90,5)		
Eficiência do sistema	I* 5 (97,7)	93,2 95,5	I* 5 (96,4)	94,8 90,5	Rapidez na execução das tarefas estabelecidas
	I* 6 (95,5)		I* 6 (90,5)		
	I* 8 (86,4)		I* 8 (97,6)		
Inconsistências	I* 6 (95,5)		I* 6 (90,5)		Ausência de erros
Facilidade de memorização	I* 2 (92,0)	92,0	I* 2 (86,9)	86,9	Sistema de fácil execução mesmo após um longo período sem utilizá-lo
Satisfação do usuário	I* 1 (89,8)	95,8	I* 1 (94,0)	94,4	*Design* agradável
	I* 4 (100,0)		I* 4 (94,0)		
	I* 9 (97,7)		I* 9 (95,2)		

* I = Item

Os resultados revelaram que todos os itens, desde a primeira versão, obtiveram escore maior que 86 nas características de usabilidade do aplicativo. Isso indica que todos alcançaram índices classificados como melhor alcançável, tornando-o uma tecnologia educacional com elevada facilidade de conhecimento e memorização do sistema, além de alta satisfação e eficiência, e de baixa inconsistência.

## Discussão

O estudo alcançou satisfatoriamente seu objetivo de construir, validar e avaliar um aplicativo móvel sobre práticas assistenciais humanizadas para recém-nascidos de risco habitual na maternidade. O aplicativo é direcionado aos profissionais de saúde, gestantes, puérperas e familiares de recém-nascidos, mediante o índice de concordância alcançado sobre conteúdo, aparência, motivação e usabilidade, tanto por item quanto global, acima dos valores desejáveis. Além disso, a usabilidade, desde a primeira versão, atingiu um nível classificado como “melhor alcançável”, indicando um excelente potencial de uso pelo público-alvo.

 É importante ressaltar a importância da criação, validação por experts e avaliação junto ao público-alvo de tecnologias educacionais em saúde, especialmente aquelas relacionadas a assuntos de interesse da população e alinhadas às suas necessidades de aprendizado ^(29)^. Pelo uso desses produtos tecnológicos, é possível aumentar o acesso a informações de qualidade em saúde, promovendo a autonomia e empoderamento tanto dos profissionais de saúde quanto das famílias na promoção de boas práticas de cuidado ao recém-nascido de risco habitual na maternidade. 

 Com base em evidências científicas, percebe-se o desenvolvimento global de tecnologias educacionais em formato de aplicativos móveis, com o propósito de instruir, informar e orientar. Um estudo criou e avaliou um *Serious Game* com foco no aprimoramento do cuidado com bebês prematuros por seus familiares, obtendo resultados convergentes com o presente estudo. A avaliação do jogo revelou níveis satisfatórios de conteúdo, aparência e usabilidade, além de estimular os participantes no processo de aprendizado ^(29)^. 

 Outro estudo elaborou, validou e avaliou uma tecnologia sobre a ordenha do leite materno destinada a enfermeiros do trabalho em agroindústrias. Essa pesquisa validou o Índice de Validade de Conteúdo (IVC) com especialistas em 86,72% e avaliou a usabilidade do jogo em forma de aplicativo por esses enfermeiros, obtendo um escore de 83,89 ^(30)^. Esses índices foram inferiores aos apresentados no presente estudo, que utilizou a mesma escala de avaliação. Outra investigação desenvolveu e validou um aplicativo móvel para gestantes em acompanhamento pré-natal abarcando questões sobre aleitamento materno. Esse estudo alcançou entre experts um IVC de 0,89, demonstrando confiabilidade nas informações e na parte técnica do sistema. Esse aplicativo apresenta potencial como recurso para promover a saúde durante o período gravídico-puerperal ^(13)^.

 Um projeto criou o aplicativo “*Preemie Care*” para orientar o cuidado neonatal por profissionais de saúde, avaliando sua usabilidade com especialistas por meio da escala SUS, alcançando um escore de 88%. Isso indicou uma usabilidade geral aceitável e excelente ^(18)^, aproximando-se do atual estudo, que obteve classificação “melhor alcançável” em todas as características do sistema. 

 No processo de construção, validação e avaliação do aplicativo “Descomplicando a amamentação”, as etapas de validação e avaliação foram conduzidas em estudos separados. Na validação de conteúdo, aparência e usabilidade utilizando o IVC, obteve-se uma média de 0,96 ^(22)^. Na avaliação da semântica, aparência, e usabilidade através do IC e da escala SUS, respectivamente, foram alcançados valores de 0,99 e 93, indicando resultados satisfatórios e altamente eficazes para uso pelas famílias ^(31)^. Esses resultados são semelhantes aos obtidos na presente pesquisa, que foram superiores em alguns aspectos. 

 Apenas uma pesquisa encontrada realizou a validação e a avaliação no mesmo estudo ^(30)^. Apesar de todos os itens terem alcançado excelentes resultados, a presente investigação obteve desempenho superior tanto na validação com experts quanto na avaliação com o público-alvo. Vale destacar que a maioria dos aplicativos móveis localizados destinados a essa população se concentra em gestações de risco e nos cuidados específicos aos recém-nascidos prematuros. No entanto, o diferencial do presente estudo reside na abordagem voltada para gestações e recém-nascidos de risco habitual, proporcionando, assim, uma contribuição significativa no contexto da saúde materno-infantil. 

 Estudos internacionais demonstraram que o uso de aplicativos móveis acerca da gestação, parto e nascimento são eficientes na melhoria do conhecimento de profissionais de saúde e familiares trazendo informação e empoderamento ao usuário ^(32 - 33)^. Podem, ainda, apoiar a comunicação entre as famílias e os profissionais durante o período pré-natal, fortalecendo a tomada de decisões ^(34)^, reforçando a importância do estudo atual. 

 Para que essas tecnologias sejam eficazes, é fundamental considerar aspectos essenciais que determinam a qualidade de um *software*, como avaliado pela escala SUS neste estudo. Essa escala abrange vários aspectos do sistema, como interface, suporte necessário, complexidade, entre outros. Portanto, essa ferramenta de avaliação apresenta elevada validade na mensuração da usabilidade de um aplicativo, configurando-se como um instrumento de avaliação confiável e robusto ^(27)^.

 Essa classificação avaliou o aplicativo móvel “Nasci Bem” como um dispositivo com usabilidade melhor alcançável, retratando fácil utilização, satisfação, eficiência e baixa inconsistência percebidas pelos usuários. Logo, os testes de usabilidade se tornam essenciais antes de disponibilizar um aplicativo para o público, sendo um dos principais critérios para garantir que um aplicativo móvel seja de fácil utilização pelos usuários e alcance seus objetivos propostos ^(35)^.

As limitações do estudo incluem a seleção por conveniência, restringindo a análise àqueles participantes selecionados e a dependência de acesso à internet e dispositivos móveis, ressaltando a necessidade de estudos adicionais com diferentes públicos e outras abordagens metodológicas.

A disponibilização do aplicativo “Nasci Bem” pode reduzir práticas inadequadas, como separação da mãe e do bebê ao nascimento, clampeamento precoce do cordão umbilical e aspiração das vias aéreas e gástrica na sala de parto, bem como a adoção de práticas adequadas como aleitamento materno e contato pele a pele precoces. A tecnologia reúne práticas assistenciais humanizadas ao recém-nascido, abrangendo recomendações atualizadas em um único local, empoderando o público-alvo, que pode acessar essas informações a qualquer momento, promovendo maior autonomia. Busca-se, assim, reduzir a morbimortalidade neonatal e infantil e expandir a saúde digital.

## Conclusão

Os profissionais da saúde e familiares que participaram das etapas metodológicas deste estudo consideraram o aplicativo “Nasci Bem” como compreensível, relevante, pertinente, de fácil utilização, baixa inconsistência e com excelente potencial de uso como tecnologia educacional em saúde.

Essa ferramenta inovadora emerge como uma ferramenta facilitadora para profissionais de saúde, proporcionando capacitação em práticas humanizadas ao recém-nascido. Além disso, oferece suporte às gestantes, puérperas e familiares, permitindo a construção de conhecimento desde o pré-natal sobre as melhores práticas de cuidado com o bebê, tornando o aprendizado prático e acessível.

 O aplicativo está disponível para *download* gratuito no sistema operacional *Android*, na plataforma *Google Play Store*, e em breve também estará acessível para iOS, na *Apple Store*.
